# The prion protein is embedded in a molecular environment that modulates transforming growth factor β and integrin signaling

**DOI:** 10.1038/s41598-018-26685-x

**Published:** 2018-06-05

**Authors:** Farinaz Ghodrati, Mohadeseh Mehrabian, Declan Williams, Ondrej Halgas, Matthew E. C. Bourkas, Joel C. Watts, Emil F. Pai, Gerold Schmitt-Ulms

**Affiliations:** 1 Tanz Centre for Research in Neurodegenerative Diseases, University of Toronto, Krembil Discovery Centre, 6th Floor, 60 Leonard Avenue, Toronto, Ontario M5T 0S8 Canada; 2 Department of Laboratory Medicine & Pathobiology, University of Toronto, Medical Sciences Building, 6th Floor, 1 King’s College Circle, Toronto, Ontario M5S 1A8 Canada; 3 Department of Biochemistry, University of Toronto, Medical Sciences Building, 5th Floor, 1 King’s College Circle, Toronto, Ontario M5S 1A8 Canada; 4 Department of Medical Biophysics, University of Toronto, Princess Margaret Cancer Research Tower, 101 College Street, Toronto, Ontario M5G 1L7 Canada

## Abstract

At times, it can be difficult to discern if a lack of overlap in reported interactions for a protein-of-interest reflects differences in methodology or biology. In such instances, systematic analyses of protein-protein networks across diverse paradigms can provide valuable insights. Here, we interrogated the interactome of the prion protein (PrP), best known for its central role in prion diseases, in four mouse cell lines. Analyses made use of identical affinity capture and sample processing workflows. Negative controls were generated from PrP knockout lines of the respective cell models, and the relative levels of peptides were quantified using isobaric labels. The study uncovered 26 proteins that reside in proximity to PrP. All of these proteins are predicted to have access to the outer face of the plasma membrane, and approximately half of them were not reported to interact with PrP before. Strikingly, although several proteins exhibited profound co-enrichment with PrP in a given model, except for the neural cell adhesion molecule 1, no protein was highly enriched in all PrP-specific interactomes. However, Gene Ontology analyses revealed a shared association of the majority of PrP candidate interactors with cellular events at the intersection of transforming growth factor β and integrin signaling.

## Introduction

Relatively little is known about how interactions of a given protein differ across cell models. Although there is no shortage of proteins whose binding partners have been studied in more than one paradigm, chances are such studies were done by separate investigators with different methodologies, precluding robust conclusions on the confounding effects of the paradigm itself.

In particular, proteins like the cellular prion protein (PrP^C^), which lack catalytic domains and exhibit widespread expression^1–3^, are prone to escape robust functional assignments. PrP^C^ is central to the pathogenesis of prion diseases^4^ and has been proposed to also act as a critical cell surface receptor in Alzheimer’s disease (AD)^5^, raising the expectation that insights into the function of PrP^C^ will provide useful angles for understanding the molecular underpinnings of these diseases.

To this end, the molecular interactions of PrP^C^ have repeatedly been characterized^6–8^ and many proteins have been reported to interact with PrP^C^ in separate studies, including the laminin receptor precursor^9^, the neural cell adhesion molecule 1 (Ncam1)^7^, the amyloid precursor like protein-1^10^, and the stress-inducible protein 1^11^. Rather than suggest a common theme, this line of investigation has led to many hypotheses regarding the role of PrP^C^. Although it is to be anticipated that some of the reported interactions will not stand the test of time, other reasons for the diversity of observations need to be considered, including the likely existence of cell type-specific interactions.

In one study undertaken with neuroblastoma cells, Zrt- Irt-like proteins (ZIPs), a family of Type-III transmembrane proteins known to import zinc and other divalent cations into the cytosol, were initially observed as PrP^C^ interactors^6^. This work then spurred the discovery that prion genes evolved from an ancient ZIP transporter and are members of the ZIP gene family, which comprises seventeen genes in humans^12,13^. Because PrP is homologous to the ectodomain present in a subset of ZIPs, studying the physiological function of this ectodomain may provide additional hints regarding PrP’s function. Interestingly, the deficiency of PrP, Zip6 or Zip10 causes a rare and phenotypically indistinguishable gastrulation arrest phenotype in zebrafish, apparently due to the ablation of a morphogenetic program known as epithelial-to-mesenchymal transition (EMT)^14–16^.

We recently documented that the expression levels of the aforementioned ZIPs, PrP, and Ncam1 are several-fold upregulated during EMT in mammalian cells^17^, consistent with the interpretation that the interactions these proteins engage in change over time and depend on the cell lineage characteristics of the model. A hint that there may be additional differences in PrP interactions when comparisons are done across models came from an observation of model-dependent proteome shifts in PrP-deficient cells^18^. Not only did PrP-deficiency in distinct cell models cause the levels of members of the Marcks protein family to shift in opposite ways but it also prevented Ncam1 polysialylation in one cell model, yet caused a robust increase of this specific posttranslational modification (PTM) in another model^17^.

To address if these phenotypic differences reflect distinct, immediate PrP interactions or depend on downstream signaling, we undertook deep, quantitative PrP interactome comparisons in four cell models that made use of *in vivo* crosslinking and capitalized on recombinant Fabs for the selective capture of endogenous PrP-containing protein complexes. We identified cell lineage-specific sets of PrP interactors, including several novel interactors and a membrane protein of unknown function, whose PrP-dependent capture was supported by high-confidence peptide-to-spectrum assignments and quantitation. All of the 26 candidate PrP interactors we identified are known to be embedded in the plasma membrane or exist in the lumen/extracellular matrix. We demonstrate that Ncam1 is the only transmembrane protein that is a universal and robust interactor of PrP across the four models. The data comprise examples of homologous proteins interacting with PrP in a cell model-specific manner. Finally, we highlight that a majority of PrP interacting proteins observed in this study are known to play roles in EMT, either by acting as transforming growth factor β (Tgfb1) signaling modulators, by facilitating the formation of Ncam1-dependent focal adhesion complexes, or by their association with integrin-mediated downstream cell signaling.

## Results

### Design of comparative PrP interactome analysis in four mouse cell models

**Figure 1:**
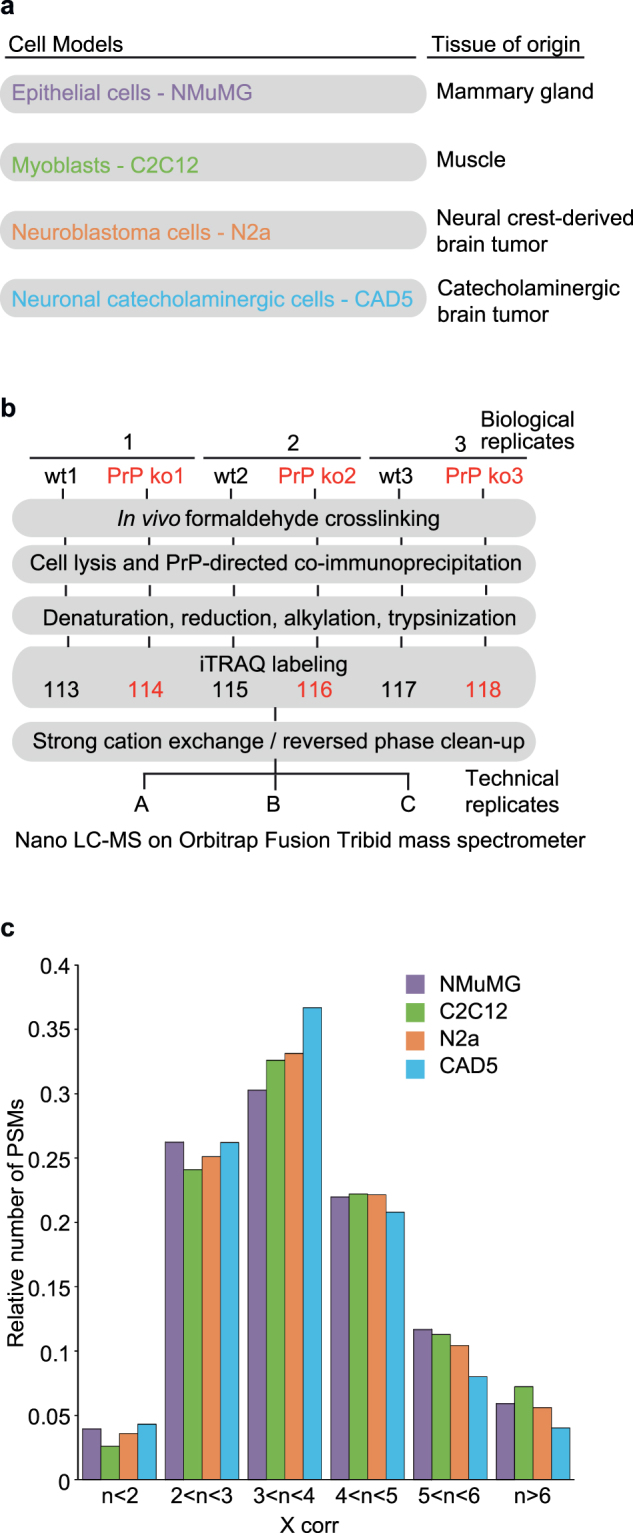
Design of comparative PrP interactome study. (**a**) Models used in this study. (**b**) Flow-chart depicting workflow of quantitative interactome analyses. Note that for the NMuMG cell model, one additional step was inserted into the workplan, namely a 48-hour addition of Tgfb1 to the cell culture medium, which causes the cells to acquire mesenchymal morphology. (**c**) Similar SEQUEST X corr stratifications of the four cell type-specific PrP interactome datasets indicated comparable data quality.

The study made use of four mouse cell lines from which we had previously derived PrP knockout clones by CRISPR-Cas9 technology (Fig. 1a)^18^. The parental wild-type cell lines are familiar to the prion research community due to their distinct properties with regard to PrP: (1) NMuMG cells exhibit a more than five-fold increase in their PrP protein levels when EMT was induced by the addition of Tgfb1 (attempts to infect these cells with prions have been unsuccessful but were also not exhaustive)^19^; (2) C2C12 cells are the only muscle cell model currently known to be susceptible to prion infection^20,21^; (3) N2a neuroblastoma cells may be the most often used cell model in prion research and can readily be infected with mouse-adapted Rocky Mountain Laboratory (RML) prions; and (4) CAD5 catecholaminergic cells exhibit susceptibility to infection with several prion strains^22^. To stabilize existing protein-protein interactions, cells were subjected to mild formaldehyde crosslinking prior to cell lysis (Fig. 1b). PrP-containing complexes were affinity-captured using a recombinant anti-PrP antigen binding fragment (Fab), designated as D18, that is known to bind to a non-linear epitope (comprising mouse PrP residues 133–157) within the globular domain of PrP^23^. Affinity-capture eluates were processed using a workflow that facilitated the relative quantitation of peptides in three biological replicates and three control samples obtained from wild-type and PrP knockout cells, respectively. The side-by-side binning of peptide-to-spectrum matches on the basis of cross-correlation (*X corr*) values computed by the SEQUEST score function^24^ revealed similar stratifications for the four PrP interactome analyses, indicating that the respective datasets were comparable in regards to a key quality control benchmark and their depths of coverage (Fig. 1c).

### Comparison of PrP interactome analyses across models

**Figure 2:**
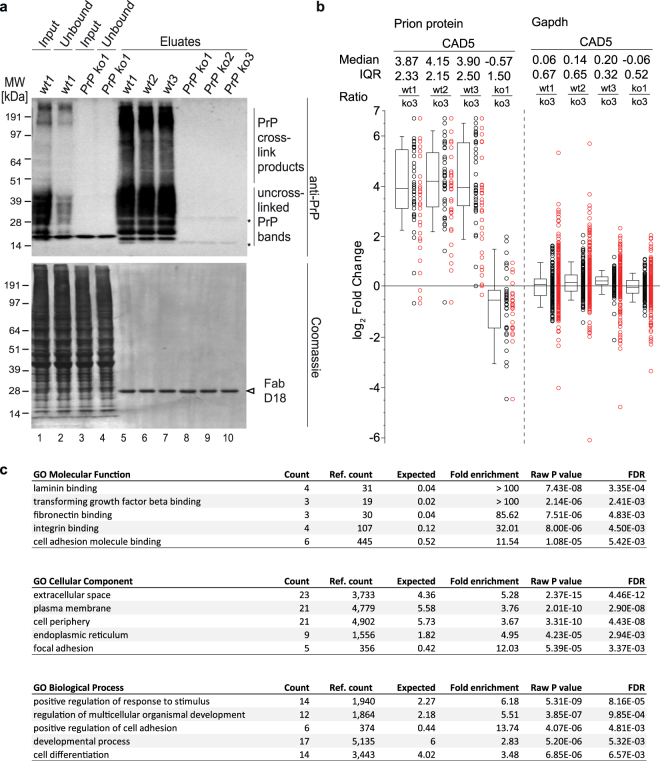
Validation of successful technical execution of quantitative interactome analysis. Analyses of benchmarks of PrP co-immunoprecipitation, iTRAQ quantitation and GO enrichment. (**a**) Western blot validation of co-immunoprecipitation of endogenous PrP^C^ from NMuMG cell extracts. Strong depletion of PrP-related signals in the unbound fraction and its robust enrichment in wild-type eluate fractions. Asterisks denote weakly detected cross-reactive bands. (**b**) Box plot depicting selective detection of PrP in wild-type eluate samples of CAD5 cell-derived PrP immunoaffinity captures but not in negative control PrP knockout eluates (see Supplementary Figure S1. for the respective PrP box plots from NMuMG, C2C12 and N2a cells). The box plot depicts in log2 space enrichment ratios of individual PrP peptides used for quantitation. The computed Median peptide ratios and Inter Quartile Ranges (IQR) are shown above the graph. Note that a subset of PSMs (indicated with red circles) were automatically eliminated from the quantitation, either because their identification was redundant or as a consequence of mass spectrometry profiles underlying their identification not passing stringency thresholds. In this and other box plots in this report relative protein levels are depicted as ratios, with ion intensities of the heaviest isobaric labels within multiplex analyses (representing one of 3 PrP knockout biological replicates) serving as the reference (denominator). (**c**) GO enrichment analyses of the 26 shortlisted PrP candidate interactors identified in this study.

A comparison of PrP-specific western blot signals obtained for cell lysates before (input) and after (unbound) the affinity capture step established that more than 50% of total PrP was captured (Fig. 2a, lanes 1 and 2). As expected, eluate fractions exhibited strong PrP-specific signals whose distribution matched the anticipated pattern, i.e., were comprised of low mass uncrosslinked bands characteristic for PrP and high mass crosslinked PrP-containing smears that were particularly concentrated in the 200–250 kDa range. When the same blot membranes were subsequently stained with Coomassie, equal total protein levels were detected in ‘input’ and ‘unbound’ samples. Eluate fractions contained protein levels below the Coomassie detection limit, except for one band whose apparent molecular weight (MW) matched the known D18 mass (Fig. 2a, lanes 5–10). The band could be seen at equal intensity levels in all biological replicates of wild-type and control samples, consistent with the interpretation that it indeed represented small amounts of the Fab, which had detached during the pH 1.9 elution step. The subsequent mass spectrometry analysis confirmed the successful enrichment of PrP (Fig. 2b, Supplementary Fig. S1).

A total of 26 proteins were shortlisted as PrP candidate interactors. Gene Ontology enrichment analyses flagged categories within the ‘Molecular Function’, ‘Biological Process’ and ‘Cellular Component’ classes that made sense for a GPI-anchored molecule like PrP (Fig. 2c). For example, a cell-to-matrix binding category, namely binding to laminin was the most enriched ‘Molecular Function’ subcategory. Similarly, 23 of the candidate interactors had a prior annotation that identified them as members of the ‘Cellular Component’ subcategory ‘extracellular space’ (see below for details on membrane topology). Finally, ‘Biological Process’ annotations of these proteins are consistent with the notion that PrP is embedded in a membrane domain that serves as a signaling hub, with the subcategory ‘positive regulation of response to stimulus’ being the most overrepresented annotation.

Although each of the four PrP interactome datasets comprised more than 200 proteins which had passed confidence thresholds for identification, approximately 80% of the proteins in each dataset were observed at levels that did not differ in wild-type versus PrP knockout samples, thereby revealing them to be non-specific interactors of the affinity matrix. An example which showcases proteins in this broad non-specific binder category is glyceraldehyde 3-phosphate dehydrogenase (Gapdh). This protein was robustly identified in all samples on the basis of dozens of peptide-to-spectrum matches (PSMs), yet was readily identified as a non-specific binder by its similar enrichment (<1.5-fold) in all samples, including in eluates derived from PrP knockout samples (Fig. 2b and Supplementary Fig. S1). Of note, rather than manifesting as a hindrance, proteins in the non-specific binder category served as a useful internal control in these analyses that further validated the existence of comparable capture conditions across all samples. A closer look at the datasets revealed one additional group of highly abundant proteins, whose enrichment behavior suggested them to be non-specific interactors. Like Gapdh, these proteins were observed in all samples, yet in contrast to Gapdh, they exhibited inconsistent and, when encountered, only modest co-enrichment with PrP. In this category fell a subset of ribosomal proteins, histones, tubulins and 14-3-3 proteins. These proteins tended to exhibit the same trend in a given sample. For example, in the second (wt2) versus third (wt3) biological replicate of the PrP interactome analysis from NMuMG cells, most of these proteins were seen at levels that were consistently lower or higher, respectively, than those observed in the corresponding PrP knockout control sample (Supplementary Table S1). The distribution of these proteins suggested them to bind mostly non-specifically to the affinity matrix but also indicated that their binding was more responsive to sample-to-sample variations than the aforementioned *bona fide* non-specific interactors.

Based solely on their iTRAQ-enrichment characteristics, the 26 PrP candidate interactors could be sorted into three categories (Table 1), namely:

(i) Proteins observed in all or a subset of the four interactome analyses that exhibited consistent and intermediate (1.5- to 7.5-fold) co-enrichment with PrP: This category encompassed the largest number of candidate PrP interactors (18 proteins), including protein disulfide isomerase (P4hb), galectin-1 (Lgals1), calreticulin (Calr), two gene products of the histocompatibility antigen gene cluster (H2-K1 and H2-D1), transmembrane emp24 domain-containing protein family members 2, 9 and 10 (Tmed2, 9 and 10), the sodium/potassium-transporting ATPase (Atp1a1) and the transferrin receptor protein 1 (Tfrc). The intermediate enrichment levels these proteins exhibited suggested that they bound, in addition to PrP, to the affinity matrix or other proteins that were non-specifically captured.
(ii) Proteins observed in a subset of samples that exhibited consistent and profound (>7.5-fold) co-enrichment with PrP (7 proteins): This category included the ZIP10 zinc transporter (Zip10), 4F2 cell-surface antigen heavy chain (Slc3a2), the large neutral amino acid transporter small subunit 1 (Slc7a5), basigin (Bsg), endothelin-converting enzyme 1 (Ece1), thrombospondin type-1 domain containing protein 7 A (Thsd7a), and insulin-like growth factor-binding protein 5 (Igfbp5).
(iii) Proteins observed in all samples that exhibited consistent and profound co-enrichment with PrP: The neural cell adhesion molecule 1 (Ncam1) was the only protein in this category.

**Table 1:**
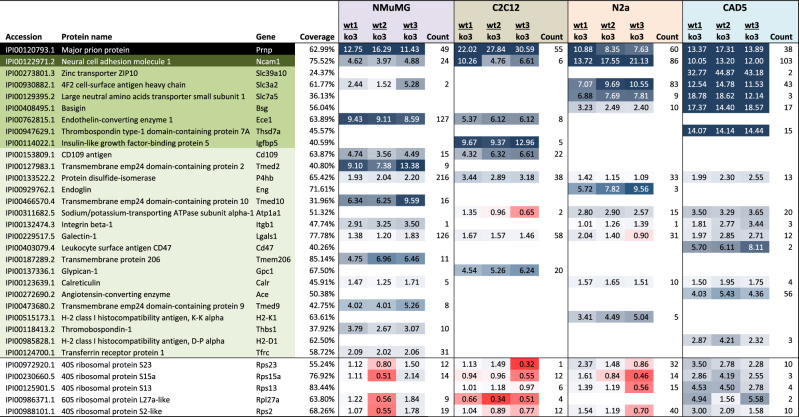
Curated list of PrP candidate interactors organized by cell type (please see Supplemental Table S1 for a complete account of shortlisted candidate interactors). Comparison of PrP interactome in four mouse cell models (list of non-specific interactors truncated, see also Supplementary Table S1). White background indicates proteins that were co-enriched in one cell model or individual biological replicates but were revealed to be non-specific interactors in other samples. Green-shading identifies PrP candidate interactors, with light green, medium intensity green and dark green designating proteins assigned to interactor categories I, II and III, respectively (see main text for details). Coverage: percent of primary structure covered by peptide-to-spectrum-matches in all four interactome analyses. Blue and red shadee numbers represent the relative average quantitations of co-purified proteins in wild-type (wt) versus *Prnp* knockout (ko) control samples. The indices following the ‘wt’ and ‘ko’ label identify the biological replicate. The count columns inform about the number of relative quantitations underlying the average iTRAQ enrichment ratios for each protein and cell model.

### Cell type-specific effects of PrP knockout on the global proteome reflect its molecular interactions

**Figure 3:**
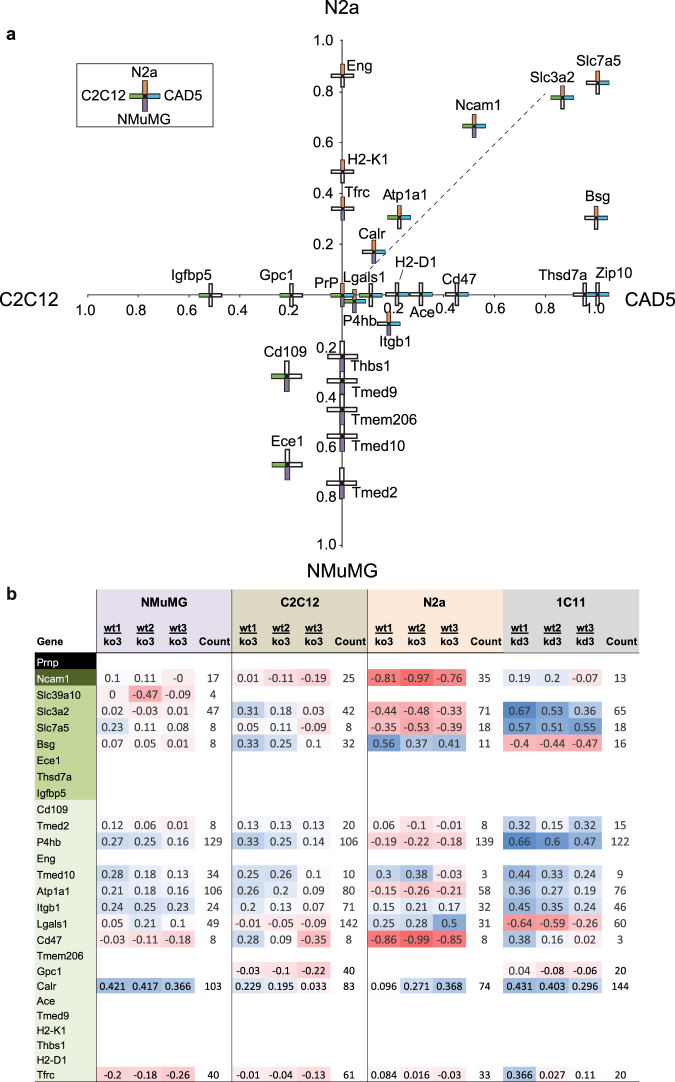
The molecular environment of PrP is cell model-specific and comprises several novel candidate interactors. (**a**) Graph depicting relative enrichment levels of candidate PrP interactors by cell type. The x-coordinate of each protein is its average WT/PrP KO ratio in CAD5 minus its average WT/PrP KO ratio in C2C12. Each PrP interactor is represented by a cross, whose position in the coordinate system is determined by its relative enrichment in the four cell models, i.e., the y-coordinate represents the average WT/PrP KO ratios observed in N2a versus NMuMG cell PrP interactomes. The average WT/PrP KO ratios used were normalized against the average WT/PrP KO ratio for PrP in the same cell line. The cell lines in which a given protein was quantified are indicated by shading in the corresponding cross arms, proteins identified in all cell types are therefore represented by fully shaded crosses while proteins identified in only one cell type are represented by crosses with one shaded arm. At least one protein was enriched to an exceptional degree in each cell line. Eng was quantified only in the N2a interactome, and Igfbp5 was similarly quantified only in the C2C12 dataset, each protein being second only to PrP in its level of enrichment. The figure demonstrates that the two neuron-like cell lines (N2a and CAD5) share several PrP interactors which were only weakly detected or undetected in NMuMG or C2C12 datasets. Conversely Ece1 and Cd109 were highly co-enriched with PrP in NMuMG and C2C12 datasets but not N2a or CAD5 PrP interactomes. (**b**) Global proteome analyses of wild-type versus PrP-deficient cells indicates that PrP depletion has reproducible effects on steady-state protein levels of shortlisted PrP candidate interactors within a given model but leads to inconsistent consequences of their relative abundance across cell models.

We next analyzed cell type-specific differences of PrP candidate interactors (Fig. 3a), making use of a coordinate system that captured if PrP interactors were observed in a given cell model and also considered their cell model-specific levels of co-enrichment with PrP. Viewed in this manner, it was apparent that not only the identity of PrP interactors but also their levels of enrichment were most similar in N2a and CAD5 cells, with Calr, Atp1a1, Ncam1, Slc3a2 and Slc7a5 ending up in proximity to a trend line that bisected the quadrant bounded by the coordinate axes assigned to these cell models, a finding congruent with the shared neuronal origins of these cell models.

Often, next neighbor relationships of proteins are reflected in mutual effects on their expression levels. We therefore were curious to understand if and how the presence or absence of PrP might affect the steady-state expression levels of the proteins it is surrounded by in a given cell model. To answer this question, we were able to lean on data we had collected in a previous study that explored how PrP knockout affects the global proteome in an overlapping set of cell models (Fig. 3b)^18^. The data from this earlier work were based on three of the four cell models used in this study (i.e., NMuMG, C2C12 and N2a cells, however, instead of CAD5 it had explored 1C11 neuroectodermal cells) and had led to the relative quantitation of >1,500 proteins, per cell model, whose identification passed a 95% confidence threshold, including a dozen PrP candidate interactors revealed in the current study. Naturally, we were particularly interested in the possible influence of PrP deficiency on the proteins we had assigned to categories II and III on account of their PrP-specific co-enrichment. The steady-state levels of four of these proteins, namely Ncam1, Slc3a2, Slc7a5, and Bsg, were quantified in all cell models on the basis of more than six independent iTRAQ signature ion ratios (counts >6) (Fig. 3b). As reported above, Slc3a2, Slc7a5 and Bsg were observed to co-immunoprecipitate with PrP on the basis of robust quantitations (counts >6) in N2a cells but not in NMuMG or C2C12 cells (Table 1). Interestingly, this cell model-specific relationship was reflected in the global proteome data, which documented that the steady state levels of these proteins were pronouncedly perturbed in response to the PrP knockout only in the N2a cell model, and were only mildly affected in NMuMG cells or C2C12 cells (Fig. 3b). This correlation was observed despite the fact that the steady-state levels of these proteins across the cell models were similar. Interestingly, the direction of change observed in N2a cells was not consistent, i.e., whereas Bsg steady-state levels increased in the absence of PrP, levels of Slc3a2 and Slc7a5 were diminished. Taken together, this result corroborated the notion that the presence of PrP has a modulating effect on the steady-state levels of proteins in its immediate proximity.

### PrP selectively interacts with Ece1 and Tfrc dimers

**Figure 4:**
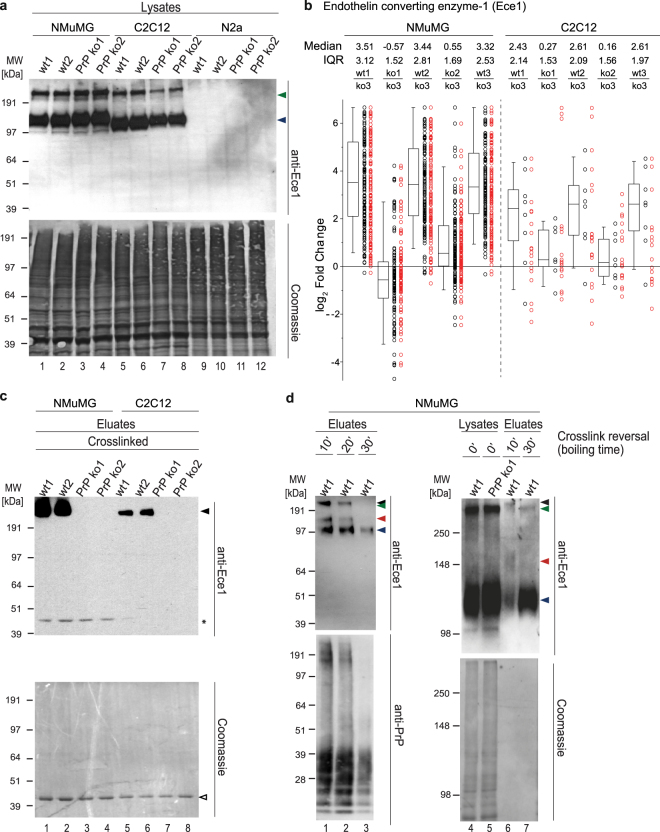
PrP interacts selectively with the Ece1 dimer, not its more abundant monomer. (**a**) Ece1-specific western blot analysis of cellular extracts generated from wild-type and PrP knockout cell models (each sample shown with two biological replicates). Cells were subjected to mild formaldehyde crosslinking prior to their harvest. Arrowheads indicate signals derived from monomeric and SDS-stable dimeric Ece1. Note that consistent with the identification of Ece1 as a PrP interactor in NMuMG and C2C12 cells, but not in N2a cells, the protein is not observed in the latter cell model. The bottom panel depicts a Coomassie stain of the western blot membrane. (**b**) Box plot depicting relative quantitation of Ece1 in PrP interactome datasets of *in vivo* formaldehyde crosslinked wild-type and PrP ko NMuMG and C2C12 cells. Please see legend to Fig. 2b for a detailed description of graph elements. (**c**) PrP co-immunoprecipitation led to the co-enrichment of a slow migrating Ece1 antibody-reactive band in wild-type (but not in PrP knockout cell) eluates, suggestive of a selective interaction of PrP with the Ece1 dimer. The asterisk denotes a non-specific cross-reactivity of the antibody, most likely toward a component of the affinity matrix that is indicated with an empty arrowhead in the Coomassie stain depicted in the bottom panel. (**d**) Formaldehyde crosslink reversal treatment (90 °C, for 10–30 minutes) causes high mass Ece1 antibody-reactive band to shift to faster gel migration at level of monomeric Ece1. Note that the panels on the left (Lanes 1–3) and right (Lanes 4–7) were generated with SDS-PAGE gel systems of different resolution. Portions of the Ece1 western blots that showed no signals were cropped at the bottom and the corresponding Coomassie stains in this panel were trimmed accordingly. Arrowheads provide signal interpretation as follows: black, Ece1 dimer-PrP complex; green, Ece1 dimer; red, Ece1 monomer-PrP complex; blue, Ece1 monomer.

In addition to several previously known PrP interactors (Ncam1, Slc3a2, Slc7a5, Bsg, P4hb, Itgb1, Lgals1, Tmem206, Gpc1, Calr, H2-K1 and H2-D1)^6^, about half of the candidate interactors revealed in this work were not previously proposed to reside in immediate proximity to PrP. Amongst these were a few proteins that highly selectively co-purified with PrP in a subset of cell models, including Ece1, Thsd7a and Igfbp5. In particular, Ece1 stood out by a remarkable 127 count of quantified PSMs in PrP-specific immunoprecipitation eluates from NMuMG cells. The protein was also detected, albeit to a lesser extent, in the PrP interactome derived from C2C12 cells but not from N2a or CAD5 cells. Ece1 is a Type II transmembrane protein that is best known for its endoproteolytic conversion of inactive big endothelin-1 (big ET-1) to active endothelin-1 (ET-1) and belongs to the family of zinc-dependent neprilysin-related endoproteinases. A western blot analysis of selected formaldehyde crosslinked cell extracts detected the predominant monomeric Ece1 (isoform D) signals at their expected apparent MW of near 120 kDa^25^ and also revealed an SDS-resistant dimer band (most likely stabilized by the crosslinking reagent) in NMuMG and C2C12 cells (Fig. 4a). Consistent with its absence from the N2a and CAD5 interactome datasets, the Ece1-directed antibody picked up no signals for this protein in these cell models (see also Supplementary Fig. S3). The box plot of quantified spectra assigned to Ece1 exhibited the expected PrP-dependent enrichment of this protein in NMuMG- and C2C12-derived datasets (Fig. 4b). Strikingly, western blot results of eluate fractions from the interactome analyses revealed PrP to have exclusively co-immunoprecipitated the crosslinked Ece1 dimer (not the more abundant monomer) (Fig. 4c). To validate this interpretation, we next boiled aliquots of the eluate fractions for up to 30 minutes in the presence of reducing agents (a method known to revert formaldehyde crosslinks)^7^. As expected, this treatment reverted the crosslink, thereby leading to the appearance of the Ece1 monomer band through an intermediate band, which we interpreted to constitute residual levels of Ece1-monomer crosslinked to PrP (see red arrowhead in Fig. 4d). If PrP was indeed crosslinked to dimeric Ece1 in the PrP affinity capture eluate fractions, we reasoned that it might be possible to document this by separating Ece1-specific signals at higher resolution, next to Ece1 dimer bands seen in PrP knockout cell extracts. Indeed, this approach confirmed that the PrP affinity-captured Ece1 dimer migrated at a higher MW than the Ece1 dimer seen in PrP knockout cells, consistent with the interpretation that PrP had formed an SDS-resistant interaction with the Ece1 dimer in this cell model (compare levels of black and green arrowheads in the right panel of Fig. 4d).

The transferrin receptor protein 1 (Tfrc) is another protein that according to the mass spectrometry data presented in this study (Table 1) resides in immediate proximity to PrP only in the NMuMG model. In contrast to Ece1, Tfrc-directed immunoblot analyses revealed robust steady-state expression levels of this protein in cell lysates of all four models tested. Yet, an intense Tfrc immunoblot signal was only observed in PrP co-immunoprecipitation eluates from wild-type NMuMG cells (Supplementary Fig. S4). However, weak Tfrc signals were also observed in PrP-specific eluates from wild-type C2C12 and N2a cells, presumably reflecting a slightly higher sensitivity of western blot analysis over mass spectrometry-based detection for this protein.

Taken together, the Ece1 and Tfrc validation experiments indicated that the absence of a PrP-interacting protein in a cell model-specific interactome data set may reflect dramatic differences in steady-state levels (Ece1) or more subtle differences in the cellular expression of a PrP binder (Tfrc) that push the amounts of a given protein residing in immediate proximity to PrP below the level necessary for its detection.

### Candidate PrP interactors exist in the secretory pathway or at the cellular membrane

**Figure 5:**
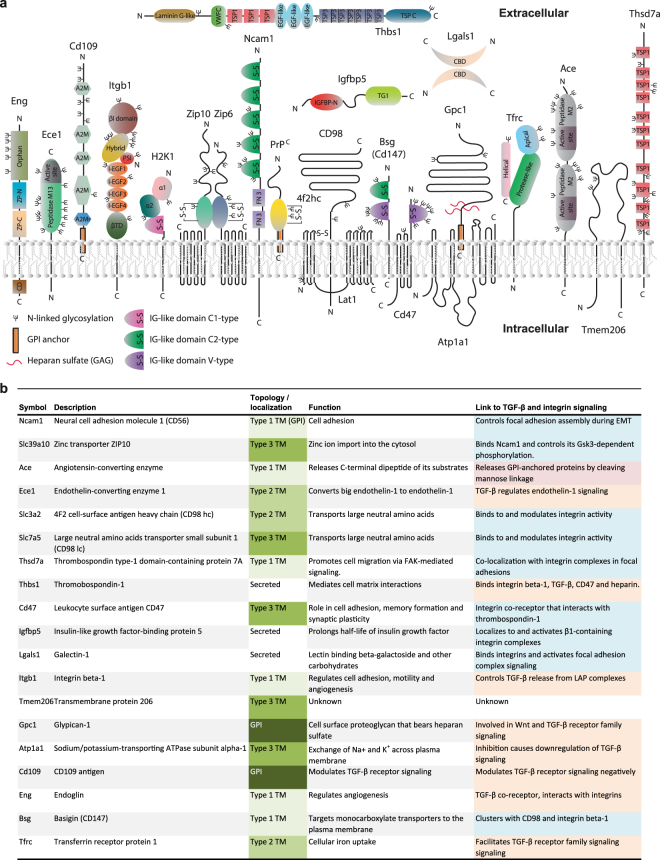
PrP’s molecular environment is enriched for proteins with known roles in Tgfb1 and integrin signaling. (**a**) Cartoon depicting domain organization, as well as known or predicted mode of membrane association of PrP interactors based on UniProt annotations. (**b**) Functional annotations of shortlisted PrP interactors.

One other way to validate interactome data is to assess the known or predicted cellular localization of proposed binders of a protein-of-interest. In light of the known, predominant association of PrP with raft-like domains at the plasma membrane^26^, PrP would be expected to encounter other proteins during its biogenesis in the secretory pathway and at the plasma membrane. A UniProt-based survey of cellular sites predicted to accommodate the identified PrP interactors established all 26 candidates as known or predicted residents of the membrane or lumen (Fig. 5), a finding consistent with the initial GO ‘Cellular Component’ enrichment analysis (Fig. 2c). Five of the candidate interactors (Calr, P4hb, as well as Tmed 2, 9 and 10) are predominantly found in the ER or Golgi, where they play critical roles for quality control and transport. Two, namely H1-K1 and H1-D1, play roles in antigen presentation and are therefore, context-dependently found in the Golgi or at the plasma membrane. Amongst the remaining 19 proteins, there are three secreted proteins, six Type-I, three Type-II and five Type III transmembrane proteins, as well as two GPI-anchored proteins. Of the latter, glypican-1 (Gpc1) is a well-known PrP interactor, that has been shown to promote both PrP’s association with detergent-resistant raft domains and its conversion to PrP^Sc ^^27–29^, but CD109 (Supplementary Fig. S2) was to our knowledge not previously reported to reside in proximity to PrP.

One approach to learn about the function of a protein-of-interest is to deduce it from the known functions of the proteins it partners with. A survey of known key functions of the 19 plasma membrane-associated PrP candidate interactors revealed their involvement in a multitude of biological activities, including cell adhesion, zinc import, endothelin-1 cleavage, transport of neutral amino acids, insulin-growth factor signaling, angiogenesis and iron uptake. Strikingly however, most of these proteins are known to also play critical roles (see Discussion for details) in either Tgfb1- or integrin-signaling (Fig. 5b).

### Tgfb1 profoundly affects steady-state levels of several PrP interactors but depletion of PrP only reduces Ncam1

**Figure 6:**
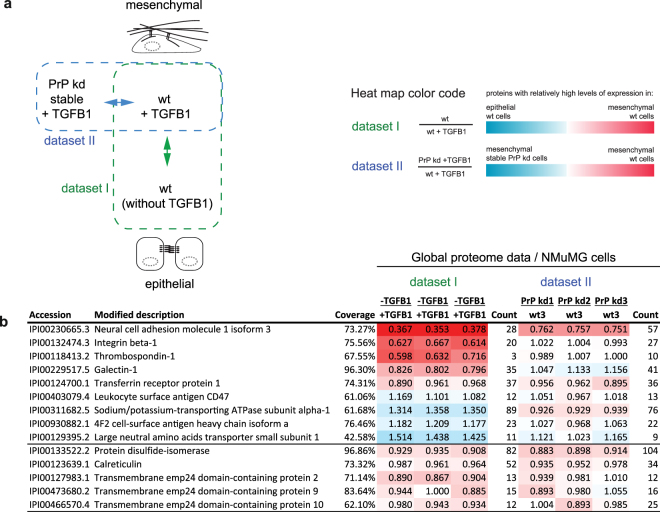
Whereas Tgfb1 treatment causes divergent shifts in steady-state levels of a subset of PrP interactors, PrP-depletion in the same paradigm only affected Ncam1 protein levels. (**a**) Schematic depicting nature of global proteome datasets mined for this study. A more complete presentation of these datasets had been published before^17^. (**b**) Comparison of steady-state protein levels of a subset of PrP interactors before and after Tgfb1 induced mesenchymal differentiation. The steady-state levels of proteins with predominant localization in the ER/Golgi compartments did not change upon two-day exposure of NMuMG cells to Tgfb1 (bottom half of table). However, proteins PrP is expected to be surrounded by at the plasma membrane underwent divergent changes in their steady-state levels. The stable PrP knockdown did not affect the protein levels of PrP candidate interactors, except for Ncam1, whose levels were diminished in PrP-deficient cells. Please see Supplementary Table S2 for a more complete presentation of underlying quantitations.

Tgfb1 had previously come to the fore in relation to PrP, because this cytokine is understood to be a major inducer of EMT, which leads cells to acquire mesenchymal morphology and to shift their mode of attachment to focal adhesion complex-based cell-matrix contacts. A link to EMT was initially suggested by the aforementioned gastrulation arrest phenotype observed in zebra fish deficient for a PrP ortholog and was recently strengthened, when we observed mammalian PrP to be dramatically induced during EMT^17^^,^^19^. Here, we were interested in how the execution of this morphogenetic program affects steady-state levels of PrP candidate interactors and in learning to what extent PrP depletion influences changes in their steady-state levels. To address these questions, we capitalized on our access to previously reported global proteome data^17^ of wild-type NMuMG cell extracts collected two days following mock- or Tgfb1-treatment (dataset I). A 2^nd^ dataset which compared the global proteomes of Tgfb1-treated wild-type cells versus Tgfb1-treated PrP-deficient cells (dataset II)^17^ could be harnessed to elucidate the effect of PrP on its nearest neighbors (Fig. 6a). Of the 26 PrP candidate interactors revealed in the current study, 14 had been quantified in dataset I on the basis of more than three PSMs with associated iTRAQ reporter ion ratios. A closer look at the relative quantitation results led us to observe that except for Ece1, Cd109 and Tmem206 (Supplementary Fig. S2), all NMuMG-cell based PrP-candidate interactors had been quantified in the global proteome analyses (datasets I and II), revealing a striking dichotomy: Whereas the steady state levels of PrP candidate interactors with predominant plasma membrane localization were affected by the Tgfb1 exposure of cells, levels of the five PrP candidate interactors with predominant residence in the secretory pathway were not (Fig. 6b). Even more strikingly, the orientation of change, i.e., whether Tgfb1 addition resulted in an increase or decrease of a given interactor, was profoundly divergent, with Ncam1, Itgb1, Lgals1 and Tfrc levels increased upon Tgfb1 exposure but levels of Cd47, Atp1a1 and subunits of the large neural amino acid transporter diminished upon exposure. When comparing PrP-depleted versus wild-type cells (dataset II), a selective reduction in Ncam1 steady-state levels was observed in contrast to the other PrP candidate interactors which showed no steady state changes. The results from these global proteome analyses speak to an intricate level of re-organization of the microenvironment of PrP during EMT (as opposed to mass effects influencing next neighbors in the same way) but also established that PrP depletion during this cellular program, except for its destabilizing effect on Ncam1 steady-state levels, has surprisingly little influence on the levels of other proteins it is surrounded by.

## Discussion

We set out to investigate the causes for the notorious lack of overlap in the primary literature concerned with the identification of PrP interactors. To this end, we made use of four mouse cell models for which PrP knockout control lines were available, using identical tools and workflows to capture endogenous PrP and its co-purifying partners. The study uncovered 26 proteins that exhibit selective or partial co-enrichment with PrP. All of these PrP candidate interactors were known or predicted to reside in cellular locations PrP is understood to have access to, and about half of them had no previous occurrence in the PrP literature, including the metalloprotease Ece1 and the GPI-anchored molecule Cd109. Except for Ncam1, we observed none of the most co-enriched proteins in proximity to PrP in all cell models. Cumulatively, our results provide a powerful testament to the conclusion that the molecular environment of PrP is to a considerable degree cell type-specific. Yet, the data also establish that across all four cell models PrP is embedded in a specialized molecular environment that appears to be tasked with governing the crosstalk of Tgfb1 and integrin signaling.

To the best of our knowledge, this is the first report that systematically compares the molecular environment of PrP in more than one cell model. A conceptual advance in this study, relative to earlier reports, including our own work^6^, was the targeting of endogenous PrP—as opposed to affinity-tagged or overexpressed protein—combined with the harnessing of PrP knockout models for the generation of negative control samples. On the basis of comparable numbers of PSMs (a parameter known to correlate with protein quantity) assigned to PrP, as well as comparable PrP sequence coverages of biological replicates within and across the four interactome datasets, it can be concluded that similar amounts of this bait protein were present in the capture eluates of all cell-type specific interactome analyses. It is therefore remarkable that the levels of protein subsets which co-purified specifically with PrP differed dramatically across the cell models. A striking example of a protein that exhibited uneven co-enrichment with PrP in a cell-type specific manner was Ece1, which was not at all observed in N2a- or CAD5-based PrP interactome datasets, yet was identified and quantified on the basis of >100 PSMs and a cumulative 63.89% sequence coverage in the NMuMG cell model. We documented that this high level of Ece1 enrichment in the NMuMG cell model was not the consequence of an unusually high level of expression in this model, as Ece1 remained below the level of detection in our global NMuMG cell proteome analyses, and as we observed similar steady-state levels for this protein in the C2C12 model by western blot analysis. Results from additional validation experiments were consistent with the interpretation that PrP was directly crosslinked to an Ece1 dimer but did not reveal reciprocal influences of Ece1 or PrP on their maturation or steady-state expression levels. Although this example validated the conceptual choice to investigate the PrP interactome in more than one model, it also exemplified the limits of conclusions that can be drawn from this study in regards to (1) previously reported PrP candidate interactors, not observed in this work, whose identification was based on other cell models or paradigms, and (2) the completeness of the current list of PrP interactors. Whenever prior reports of PrP interactors in the same paradigm were available, our current data corroborated and complemented their findings. For instance, a prior report, which documented interactors of FLAG-tagged PrP in formaldehyde crosslinked N2a cells^6^, had—with the exception of endoglin (Eng)—identified all of the PrP interactors observed in the N2a cell model in the current study. The technical improvements underlying the current study, however, helped to flag a subset of previously reported PrP candidate interactors, including several 14-3-3 proteins, tubulin and ribosomal subunits, as most likely representing non-specific interactors.

In light of the striking differences in the molecular environments of PrP in the four cell models employed in this study, it warrants repeating that Ncam1 stood out by being prominent in all PrP interactome datasets. This unique relationship of PrP and Ncam1 was further emphasized by our observation that PrP deficiency in Tgfb1 exposed NMuMG cells had no effect on the steady-state levels of the proteins found in PrP’s molecular environment, except for causing a relative depletion of Ncam1 levels. The increasingly special status of Ncam1 amongst PrP interactors corroborates previous observations: (1) formaldehyde crosslinking gives rise to SDS-stable Ncam1-PrP complexes that represent a majority of the high molecular mass PrP-crosslink signals in N2a cells^7^; (2) PrP controls Ncam1 polysialylation^17^; and (3) a recent interactome analysis of the closest PrP relatives amongst the ZIP transporters that documented a selective interaction with Ncam1 (and calreticulin) but not with other PrP interactors^30^.

Despite the distinctness of PrP interactome data in the four cell models, a Molecular Function GO term analysis pointed toward an overarching similarity in the molecular environment of PrP, apparently populated by proteins with known roles in Tgfb1 and integrin signaling. A closer look at the primary reports underlying the relevant functional annotations, as well as the broader literature, further strengthened this conclusion. For some PrP interactors, the connection to the biology of Tgfb1 and integrin signaling seems less than obvious at first. For example, Tfrc is best known as a cellular importer of transferrin-bound iron. However, robust evidence has also linked this transporter to Tgfb1 signaling (a finding that is not yet reflected in its current GO annotations). More specifically, the neural crest cell-specific knockout of Tfrc in mice caused craniofacial abnormalities, including cleft palate and micrognathia (reduced mandible), and was associated with a suppression of Tgfb1 signaling^31^. The characteristic craniofacial abnormalities observed are pathognomonic of a disease known as Pierre Robin Sequence (PRS) caused by mutations in genes that impair Tgfb1 superfamily signaling. Moreover, Cd109 is a major negative inhibitor of Tgfb1 signaling^32^ and Eng is a Tgfb1 co-receptor^33^. Notably, although these proteins share unequivocal and direct links to Tgfb1 signaling, this study did not observe their co-enrichment with PrP in the same cell model. Rather, Cd109 was detected in NMuMG- and C2C12-derived PrP interactome datasets, and endoglin was only seen in proximity to PrP in N2a cells.

Similarly, the Cd98 complex, composed of 4f2 (Slc3a2) and Lat1 (Slc7a5) subunits, is best known for its transport of large neutral amino acids. However, the primary literature and several reviews have drawn attention to the direct involvement of this complex in integrin signaling^34–36^. Cd98 exerts this involvement in part by its direct interaction with Itgb1, which is an integral component of integrin-based focal adhesion complexes and was observed in the N2a interactome dataset in this study. Additional PrP candidate interactors with connections to integrin biology were Cd47, which acts as an integrin co-receptor^37^ and was named integrin-associated protein^38^ after its discovery as a contaminant in integrin preparations, and Lgals1, which is known to bind to and activate integrin-dependent signaling of focal adhesion complexes^39–41^. As with Tgfb1-associated proteins, these integrin interactors were not all identified in the same PrP interactome dataset, but were revealed in different combinations in one or more of the cell-type specific datasets we presented. Moreover, it is increasingly understood that Tgfb1 and integrin signaling activities do not exist irrespective of one another. Rather, these signaling portals are considered sister complexes bound to each other through several connections. In fact, a subset of proteins shortlisted in this study as PrP interactors, including Cd98 and Thbs1, are known to facilitate this crosstalk^42,43^.

How does PrP influence Tgfb1 and integrin signaling complexes? By its mere presence, PrP would be expected to influence the composition and architecture of its molecular environment in many subtle ways. As PrP is a GPI-anchored molecule with affinity for membrane domains enriched in cholesterol and sphingolipids, it seems plausible that one of these influences will relate to its effect on the balance between clathrin-mediated endocytosis and caveolae-based transport. Such a balance shift is unlikely to be innocuous. For example, Tgfb1 signaling is dependent on clathrin-mediated endocytosis (references in^31^), and it has been proposed that CD109 and Tfrc influence the canonical signaling of Tgfb1 in such manner^31,32^. A similar dichotomy of internalization pathways also exists for integrin complexes and Lgals1 has been proposed to influence their trafficking^41^. A more direct influence of PrP on the regulation of focal adhesion complexes may derive from its interaction with Ncam1 and its dramatic influence on Ncam1 polysialylation^17^. Although the details of how Ncam1 promotes the establishment of cell-to-matrix contacts based on focal adhesions have remained murky, and are likely to be complex, there is ample evidence for a Tgfb1-dependent expression of Ncam1^44^ and a critical role of this protein in the molecular rearrangements that govern this cellular program^45–47^.

## Conclusion

There is a tendency to ascribe differences in interactions of a protein-of-interest reported by separate investigators mainly to differences in methodology, even when studies are undertaken with different paradigms. Insights gained in this work caution that the molecular environment of a given protein-of-interest can be surprisingly diverse when comparing distinct models. The comparative interactome data we presented placed the evolutionarily conserved interaction between PrP and Ncam1 in the context of a plasma membrane microdomain tasked with modulating the crosstalk between Tgfb1 and integrin signaling. We anticipate that further investigations will substantiate roles of PrP in cellular programs relying on these sister signaling hubs. The results from this study further suggest that, unless PrP interactome studies are undertaken with brain cells, next-neighbor relationships central to the cellular toxicity that manifests in prion disorders may be overlooked.

## Methods

### Western blot analyses

Equal amounts of protein were separated on 4–12% Bis-Tris gels (Life Technologies, Burlington, ON, Canada) and transferred to a 0.45 micron polyvinylidene fluoride membrane. The membranes were blocked with 10% skim milk in Tris-buffered saline with Tween 20 (TBST), and incubated overnight at 4 °C with the respective primary antibody. Subsequently, the western blots were washed thrice with TBST and incubated with HRP-conjugated anti-mouse (catalog number 1706516; BioRad, Mississauga, ON, Canada), anti-rabbit (catalog number 1706515; BioRad), or anti-rat (31476; Thermo Fisher Scientific, Waltham, MA, USA) secondary antibodies for two hours at room temperature. Membranes were subjected to three washes with TBST and incubated with the ECL reagent (catalog number RPN2106; Sigma Aldrich, Oakville, ON, Canada). Signals were then visualized using either X-ray film or a LI-COR Odyssey Fc digital imaging system (LI-COR Biosciences, Lincoln, NE, USA). Immunoblotting was undertaken with the Sha31 antibody against PrP (catalog number A03213; Bertin Bioreagent, Montigny le Bretonneux, France), or antibodies directed toward Ece1 (catalog number ab71829; Abcam, Cambridge, United Kingdom) or Tfrc (catalog number ab84036; Abcam).

### Cell culture and transfection

Mouse mammary gland NMuMG cells (catalog number CRL-1636; American Type Culture Collection (ATCC), Manassas, VA, USA) and Cath.a-differentiated (CAD-2A2D5; CAD5 for short) cells were a kind gift from Dr. Jeffrey Wrana (University of Toronto, Toronto, ON, Canada) and Dr. Charles Weissmann (The Scripps Research Institute, Jupiter, FL, USA), respectively. Mouse myoblast (C2C12) cells (catalog number CRL-1772) and mouse neuroblastoma Neuro-2a (N2a) cells (catalog number CCL-131) were purchased from ATCC. Cells were cultured in D(MEM) or opti-MEM supplemented with 10% heat inactivated fetal bovine serum (catalog number 12484028; Invitrogen Canada, Burlington, ON, Canada), 1% GlutaMAX (catalog number 35050061; Invitrogen Canada), and 1% antibiotic-antimycotic solution (catalog number 15240062; Invitrogen Canada). Human insulin solution (catalog number I9278; Sigma-Aldrich, Oakville, ON, Canada) was added at a concentration of 10 μg/mL for NMuMG cells.

To induce EMT, NMuMG cells were treated with Tgfb1 (catalog number 240-B; R&D Systems, Minneapolis, MN, USA) at a concentration of 6.4 ng/mL daily for 48 hours. C2C12 myoblasts were differentiated to myotubes by replacing the DMEM containing 10% FBS with medium supplemented with 2% horse serum.

The knockout of *Prnp* in NMuMG, C2C12, and N2a cells had been achieved using CRISPR-Cas9 genetic engineering and was described previously^48^. The CAD5 PrP knockout line was made with identical sgRNA reagents as the other PrP knockout lines but had not been previously described.

### Sample preparation for immunoprecipitation analyses

Comparative interactome analyses were performed with three biological replicates for each condition (unless indicated otherwise), that were side-by-side expanded to large scales (average yield of 6 × 10^7^ cells). Cells were washed with ice-cold PBS, and mildly crosslinked with 2% formaldehyde in PBS for 15 minutes at room temperature. Subsequently, residual crosslinking reagent was quenched during a 10 minute incubation with 125 mM glycine in PBS and cells were lysed in ice-cold buffer consisting of 150 mM Tris (pH 8.3), 150 mM NaCl, 0.5% NP-40, 0.5% sodium deoxycholate supplemented with protease inhibitor cocktail (catalog number 11836170001; Roche, Mississauga, ON, Canada) and phosphatase inhibitor tablet (catalog number 4906837001; Roche). Cellular debris were removed by centrifugation at 2,000 RPM for 5 minutes, followed by 30 minutes at 4,000 RPM (4 °C). Centrifugation supernatants were collected and protein levels were adjusted using a bicinchoninic acid colorimetric assay with reagents A (catalog number 23228) and B (catalog number 1859078) from Thermo Fisher Scientific.

### Protein immunoprecipitation workflow

PrP was captured using the D18 antibody, a humanized recombinant Fab developed against PrP that was provided by the laboratory of Dr. Emil F. Pai. The PrP antibody was conjugated to KappaSelect beads (catalog number 17-5458-01; GE Healthcare, Oakville, ON, Canada) under gentle agitation by a turning wheel at 4 °C overnight. The affinity capture bead/antibody mixture was then equally aliquoted for individual samples and adjusted protein samples were added for an overnight capture at 4 °C. The next day, the affinity matrices were stringently washed twice with lysis buffer and twice with lysis buffer containing 500 mM NaCl to remove non-specific binders, followed by a pre-elution wash of 10 mM HEPES, pH 8. Proteins were then eluted by acidification in 0.2% trifluoroacetic acid, 20% acetonitrile.

### Nanoscale HPLC-ESI tandem mass spectrometry

Sample preparation for mass spectrometry was done as described previously^48^. Immunoprecipitation eluates were dried under vacuum then diluted in 9 M deionized urea. Reduction with tris (2-carboxyethyl) phosphine at 60 °C was followed by room temperature sulfhydryl group alkylation with 4-vinylpyridine. The urea concentration was lowered to 1.25 M in 500 mM triethylammonium bicarbonate prior to the addition of mass spectrometry-grade trypsin (catalog number 90057; Thermo Fisher Scientific). Digestion occurred at 37 °C overnight. Trypsin treated samples were covalently modified with 8-plex isobaric tags for relative and absolute quantitation (iTRAQ) (catalog number 4390811; Sciex, Concord, ON, Canada) according to the manufacturer’s protocol then mixed. Sample mixtures were purified on reversed phase resin in Bond Elut OMIX cartridges (catalog number A57003100; Agilent Technologies, Santa Clara, CA, USA) alone and in combination with strong cation exchange cartridges (catalog number A57004100; Agilent Technologies).

All sample mixtures were analyzed over a four-hour reversed phase 300 nL/min gradient on an EASY-nLC 1000-Orbitrap Fusion Tribrid mass spectrometer platform (Thermo Fisher Scientific). The analytical column was a 25 cm long Acclaim PepMap RSLC 100 of 75 μm inner diameter with 2 µm C18 particles having 100 Å pores. Each liquid chromatography-mass spectrometry run was divided into scan cycles up to 3 seconds long, each including one orbitrap precursor ion MS scan and as many linear ion trap product ion (MS2) scans and orbitrap MS3 scans as possible within the 3 second time window. Collision induced dissociation (CID) and higher energy collisional dissociation (HCD) were used for MS2 and MS3 respectively. The Orbitrap resolution was set to 60,000 for both MS and MS3.

### Protein identification and quantification

MS2 data were converted to protein sequence information with Proteome Discoverer (version 1.4.0.288; Thermo Fisher Scientific) using the embedded Mascot and Sequest HT search algorithms with the mouse international protein index database (version 3.87). Up to two missed cleavages were allowed per peptide. The allowed peptide mass range was 400–6000 Da, with a precursor ion mass tolerance of 20 ppm and product ion mass tolerance of 0.4 Da. Variable modifications considered were asparagine and glutamine deamidation, methionine oxidation as well as serine, threonine and tyrosine phosphorylation. Cysteine pyridylethylation as well as iTRAQ 8-plex reagent labeling of peptide N-termini and lysines were defined as fixed modifications. False discovery rate estimation based on q-Value was performed with the Percolator algorithm. Relative protein quantification was produced from MS3 data by the Reporter Ions Quantifier built into Proteome Discoverer with the most confident centroid under a mass tolerance of 20 ppm.

### Data availability

The mass spectrometry proteomics data have been deposited to the ProteomeXchange Consortium via the PRIDE partner repository^49^ with the dataset identifier PXD008781.

## Electronic supplementary material

Supplementary Information

## References

[1] Basler K (1986). Scrapie and cellular PrP isoforms are encoded by the same chromosomal gene. Cell.

[2] Puckett C, Concannon P, Casey C, Hood L (1991). Genomic structure of the human prion protein gene. Amer J Hum Genet.

[3] Saeki K, Matsumoto Y, Hirota Y, Onodera T (1996). Three-exon structure of the gene encoding the rat prion protein and its expression in tissues. Virus Genes.

[4] Prusiner SB (1982). Novel proteinaceous infectious particles cause scrapie. Science.

[5] Lauren J, Gimbel DA, Nygaard HB, Gilbert JW, Strittmatter SM (2009). Cellular prion protein mediates impairment of synaptic plasticity by amyloid-beta oligomers. Nature.

[6] Watts JC (2009). Interactome analyses identify ties of PrP and its mammalian paralogs to oligomannosidic N-glycans and endoplasmic reticulum-derived chaperones. PLoS Pathog.

[7] Schmitt-Ulms G (2001). Binding of neural cell adhesion molecules (N-CAMs) to the cellular prion protein. J Mol Biol.

[8] Rutishauser D (2009). The comprehensive native interactome of a fully functional tagged prion protein. PLoS One.

[9] Rieger R, Edenhofer F, Lasmezas CI, Weiss S (1997). The human 37-kDa laminin receptor precursor interacts with the prion protein in eukaryotic cells. Nat Med.

[10] Yehiely F (1997). Identification of candidate proteins binding to prion protein. Neurobiol Dis.

[11] Zanata SM (2002). Stress-inducible protein 1 is a cell surface ligand for cellular prion that triggers neuroprotection. Embo J.

[12] Schmitt-Ulms G, Ehsani S, Watts JC, Westaway D, Wille H (2009). Evolutionary descent of prion genes from the ZIP family of metal ion transporters. PLoS One.

[13] Taylor KM, Nicholson RI (2003). The LZT proteins; the LIV-1 subfamily of zinc transporters. Biochim Biophys Acta.

[14] Malaga-Trillo E (2009). Regulation of embryonic cell adhesion by the prion protein. PLoS Biol.

[15] Yamashita S (2004). Zinc transporter LIVI controls epithelial-mesenchymal transition in zebrafish gastrula organizer. Nature.

[16] Taylor KM (2016). Zinc transporter ZIP10 forms a heteromer with ZIP6 which regulates embryonic development and cell migration. Biochem J.

[17] Mehrabian M (2015). The Prion Protein Controls Polysialylation of Neural Cell Adhesion Molecule 1 during Cellular Morphogenesis. PLoS One.

[18] Mehrabian M (2016). Prion Protein Deficiency Causes Diverse Proteome Shifts in Cell Models That Escape Detection in Brain Tissue. PLoS One.

[19] Mehrabian, M., Hildebrandt, H. & Schmitt-Ulms, G. NCAM1 Polysialylation: The Prion Protein’s Elusive Reason for Being? *ASN Neuro* **8**, 10.1177/1759091416679074 (2016). 10.1177/1759091416679074 PMC5122176 27879349

[20] Dlakic WM, Grigg E, Bessen RA (2007). Prion infection of muscle cells *in vitro*. J Virol.

[21] Herbst A (2013). Infectious Prions Accumulate to High Levels in Non Proliferative C2C12 Myotubes. PLoS Pathog.

[22] Qi Y, Wang JK, McMillian M, Chikaraishi DM (1997). Characterization of a CNS cell line, CAD, in which morphological differentiation is initiated by serum deprivation. J Neurosci.

[23] Williamson RA (1998). Mapping the prion protein using recombinant antibodies. J Virol.

[24] Klammer AA, Park CY, Noble WS (2009). Statistical calibration of the SEQUEST XCorr function. J Proteome Res.

[25] Meidan R, Klipper E, Gilboa T, Muller L, Levy N (2005). Endothelin-converting enzyme-1, abundance of isoforms a-d and identification of a novel alternatively spliced variant lacking a transmembrane domain. J Biol Chem.

[26] Naslavsky N, Stein R, Yanai A, Friedlander G, Taraboulos A (1997). Characterization of detergent-insoluble complexes containing the cellular prion protein and its scrapie isoform. J Biol Chem.

[27] Taylor DR, Whitehouse IJ, Hooper NM (2009). Glypican-1 mediates both prion protein lipid raft association and disease isoform formation. PLoS Pathog.

[28] Cheng F, Lindqvist J, Haigh CL, Brown DR, Mani K (2006). Copper-dependent co-internalization of the prion protein and glypican-1. J Neurochem.

[29] Lofgren K, Cheng F, Fransson LA, Bedecs K, Mani K (2008). Involvement of glypican-1 autoprocessing in scrapie infection. Eur J Neurosci.

[30] Brethour D (2017). A ZIP6-ZIP10 heteromer controls NCAM1 phosphorylation and integration into focal adhesion complexes during epithelial-to-mesenchymal transition. Scientific Reports.

[31] Lei R (2016). Transferrin receptor facilitates TGF-beta and BMP signaling activation to control craniofacial morphogenesis. Cell death & disease.

[32] Li C (2016). Soluble CD109 binds TGF-β and antagonizes TGF-β signalling and responses. Biochemical Journal.

[33] Valluru, M., Staton, C., Reed, M. & Brown, N. Transforming Growth Factor-β and Endoglin Signaling Orchestrate Wound Healing. *Front Physiol* **2** (2011). 10.3389/fphys.2011.00089 PMC3230065 22164144

[34] Nguyen HT, Merlin D (2012). Homeostatic and innate immune responses: role of the transmembrane glycoprotein CD98. Cell Mol Life Sci.

[35] Deves R, Boyd CA (2000). Surface antigen CD98(4F2): not a single membrane protein, but a family of proteins with multiple functions. J Membr Biol.

[36] Yan Y, Vasudevan S, Nguyen HT (2008). & Merlin, D. Intestinal epithelial CD98: an oligomeric and multifunctional protein. Biochim Biophys Acta.

[37] Isenberg JS, Roberts DD, Frazier WA (2008). CD47: A New Target in Cardiovascular Therapy. Arterioscler Thromb Vasc Biol.

[38] Brown E, Hooper L, Ho T, Gresham H (1990). Integrin-associated protein: a 50-kD plasma membrane antigen physically and functionally associated with integrins. J Cell Biol.

[39] Moiseeva EP, Williams B, Goodall AH, Samani NJ (2003). Galectin-1 interacts with beta-1 subunit of integrin. Biochem Biophys Res Commun.

[40] Fischer, C. *et al*. Galectin-1 interacts with the {alpha}5{beta}1 fibronectin receptor to restrict carcinoma cell growth via induction of p21 and p27. *J Biol Chem* **280**, 37266-37277 (2005). 10.1074/jbc.M411580200 16105842

[41] Fortin S (2010). Galectin-1 is implicated in the protein kinase C epsilon/vimentin-controlled trafficking of integrin-beta1 in glioblastoma cells. Brain Pathol.

[42] Huang T, Sun L, Yuan X, Qiu H (2017). Thrombospondin-1 is a multifaceted player in tumor progression. Oncotarget.

[43] Adams JC, Lawler J (2011). The thrombospondins. Cold Spring Harb Perspect Biol.

[44] Roubin R, Deagostini-Bazin H, Hirsch MR, Goridis C (1990). Modulation of NCAM expression by transforming growth factor-beta, serum, and autocrine factors. J Cell Biol.

[45] Lehembre F (2008). NCAM-induced focal adhesion assembly: a functional switch upon loss of E-cadherin. Embo J.

[46] Beggs HE, Baragona SC, Hemperly JJ, Maness PF (1997). NCAM140 interacts with the focal adhesion kinasep125(fak) and the SRC-related tyrosine kinase p59(fyn). J Biol Chem.

[47] Frame MC, Inman GJ (2008). NCAM is at the heart of reciprocal regulation of E-cadherin- and integrin-mediated adhesions via signaling modulation. Dev Cell.

[48] Mehrabian M (2014). CRISPR-Cas9-Based Knockout of the Prion Protein and Its Effect on the Proteome. PLoS One.

[49] Vizcaino JA (2016). update of the PRIDE database and its related tools. Nucleic Acids Res.

