# Supplementary material for: The prion protein is embedded in a molecular environment that modulates transforming growth factor β and integrin signaling
Source: Sci Rep. 2018 Jun 5;8:8654. doi: 10.1038/s41598-018-26685-x (PMC5988664; doi:10.1038/s41598-018-26685-x)
Supplement: Supplementary file 1 — Supplementary Information [file 41598_2018_26685_MOESM1_ESM.pdf]

February 7, 2018

Supplementary Information to:

The prion protein is embedded in a molecular environment that modulates transforming growth factor β and integrin signaling

Farinaz Ghodrati[1,2,¶], Mohadeseh Mehrabian[1,2,¶], Declan Williams[1,¶], Ondrej Halgas[3], Matthew E. C. Bourkas[1,3], Joel C. Watts[1,3], Emil F. Pai[3,4], Gerold Schmitt-Ulms[1,2]*

[1] Tanz Centre for Research in Neurodegenerative Diseases, University of Toronto, Krembil Discovery Centre, 6th Floor, 60 Leonard Avenue, Toronto, Ontario M5T 0S8, Canada.

[2] Department of Laboratory Medicine & Pathobiology, University of Toronto, Medical Sciences Building, 6th Floor, 1 King's College Circle, Toronto, Ontario M5S 1A8, Canada.

[3] Department of Biochemistry, University of Toronto, Medical Sciences Building, 5th Floor, 1 King's College Circle, Toronto, Ontario M5S 1A8, Canada.

[4] Department of Medical Biophysics, University of Toronto, Princess Margaret Cancer Research Tower, 101 College Street, Toronto, Ontario M5G 1L7, Canada

¶These authors contributed equally to this work.

*Please address correspondence to: g.schmittulms@utoronto.ca.

Supplementary Figure S1. Consistent and selective enrichment of PrP contrasted to non-specific binding of Gapdh.

(a) Box plots of PrP-derived peptides in all four models. Please see legend to Fig. 2b for a detailed description of graph elements. (b) Box plots of Gapdh-derived peptides in all four models.

Supplementary Figure S2. Selective PrP co-enrichment of Cd109 and Tmem206.

Box plots of CD109 and Tmem206 in the subset of datasets, in which these proteins were robustly identified and quantified. Please see legend to Fig. 2b for a detailed description of graph elements.

Supplementary Figure S3. Evidence that Ece1 is not expressed in CAD5 cells at levels detectable by western blot analysis.

Ece1 western blot analysis of formaldehyde crosslinked lysate and eluate fractions from the PrP-directed co-immunoprecipitation of wild-type and PrP knockout CAD5 cells (analogous to data shown in Fig. 4a). The results validate the PrP interactome data (Table 1) which failed to detect Ece1 in PrP-directed co-immunoprecipitations from CAD5 cell lysates. NMuMG cell lysates were loaded as a positive control for Ece1 detection, and a Coomassie-stain of the western blot documents total protein levels in the respective lysates and eluates. Arrowheads indicate signals derived from monomeric and SDS-stable crosslinked dimeric Ece1.

Supplementary Figure S4. PrP co-immunoprecipitates Tfrc from wild-type but not PrP knockout NMuMG cell lysates.

Validation of the transferrin receptor protein 1 (Tfrc) as a PrP binder. Consistent with the PrP interactome data, Tfrc is only prominently represented in PrP co-immunoprecipitation eluate

fractions derived from formaldehyde-crosslinked wild-type NMuMG cells. However, whereas Tfrc escaped detection by mass spectrometry in PrP co-immunoprecipitation eluates derived from wild-type C2C12 and N2a cells (Table 1), weak Tfrc signals can be detected in the respective eluates by western blot analysis, presumably reflecting a slightly higher sensitivity of western blot analysis over mass spectrometry-based detection for this protein. No Tfrc was observed in PrP co-immunoprecipitation eluates from CAD5 wild-type cells. Coomassie stains of the western blot membranes are shown underneath the immunoblot panels to document protein amounts in the respective samples. Blue and green arrowheads point toward Tfrc monomer and formaldehyde-crosslinked dimer signals, respectively. A higher molecular mass band (possibly a crosslink of two Tfrc dimers) can also be detected. The empty arrowhead shown in the Coomassie images indicates the D18 recombinant Fab used for PrP-directed immunoprecipitation. Note also the subtle differences in Tfrc immunoblot signal intensities when comparing wt and PrP knockout lysates in NMuMG, C2C12 and N2a cell models, which could be indicative of molecular crosstalk between PrP and Tfrc modulating steady-state levels of Tfrc.

Supplementary Table S1. Comparison of the PrP interactome in four mouse cell models.

Supplementary Table S2. Global proteome analysis of NMuMG cells -/+ TGFB1 (datset I) in PrP-deficient and wildtype cells (dataset II).

Supplementary Figure S1

a

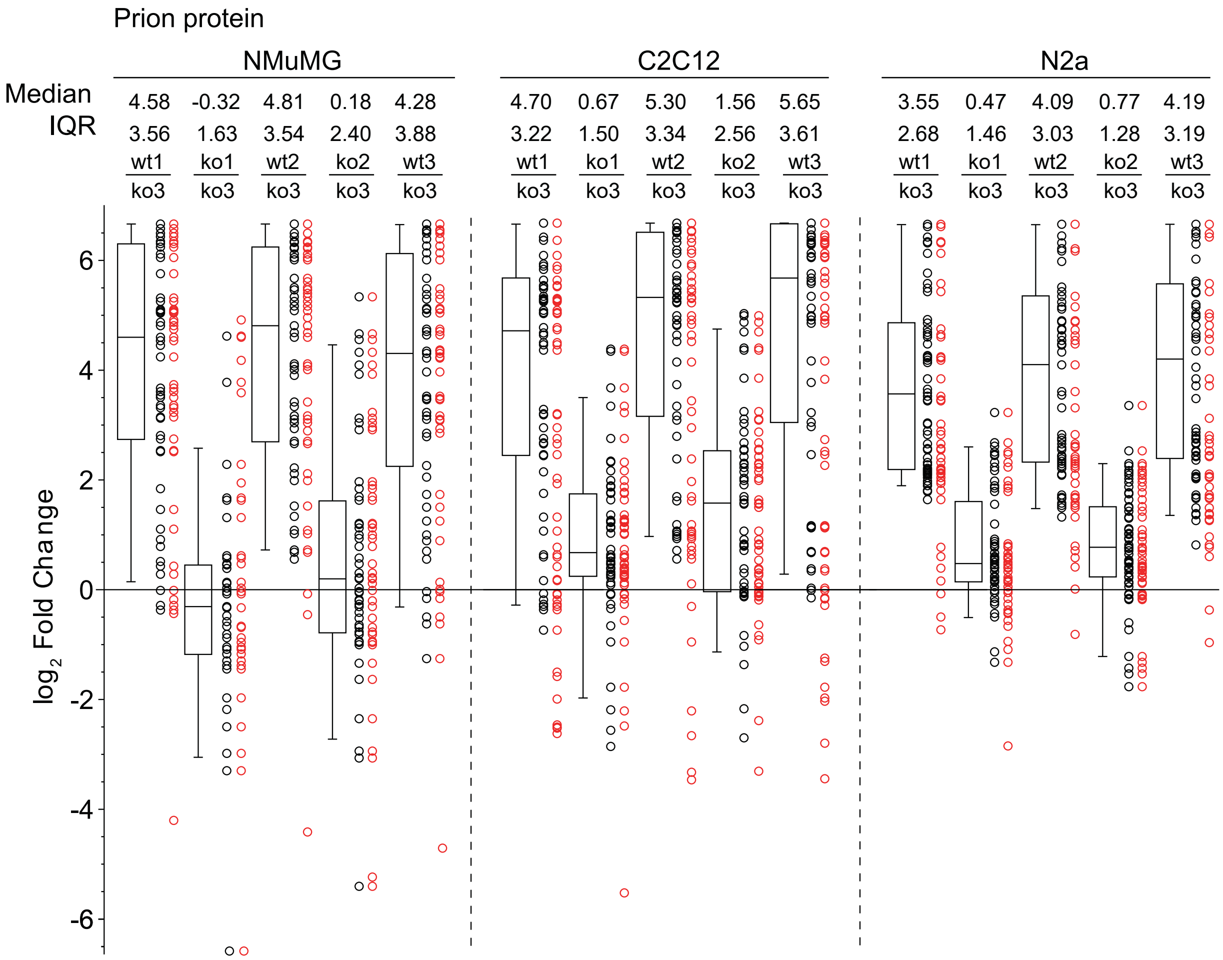

b

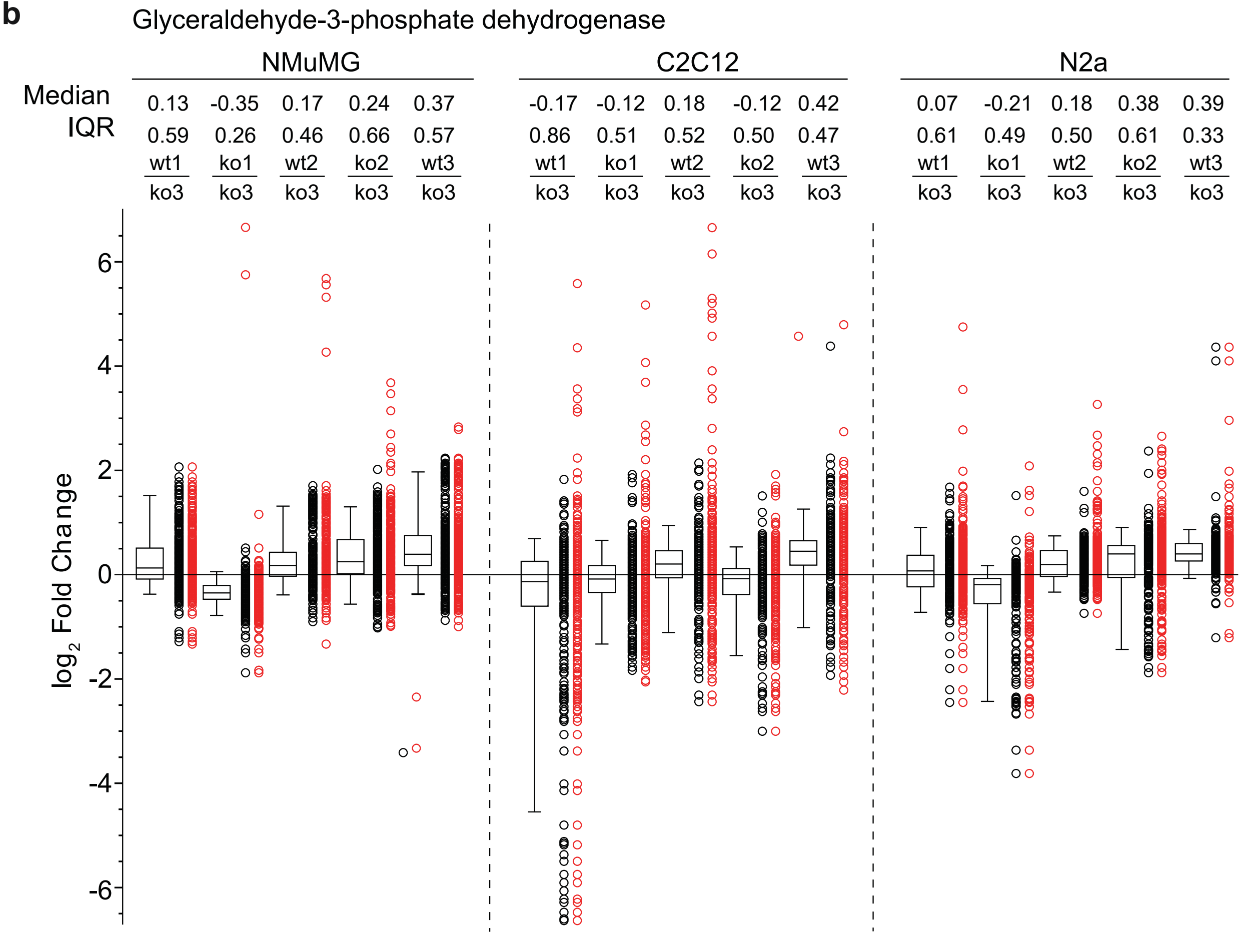

Supplementary Figure S2

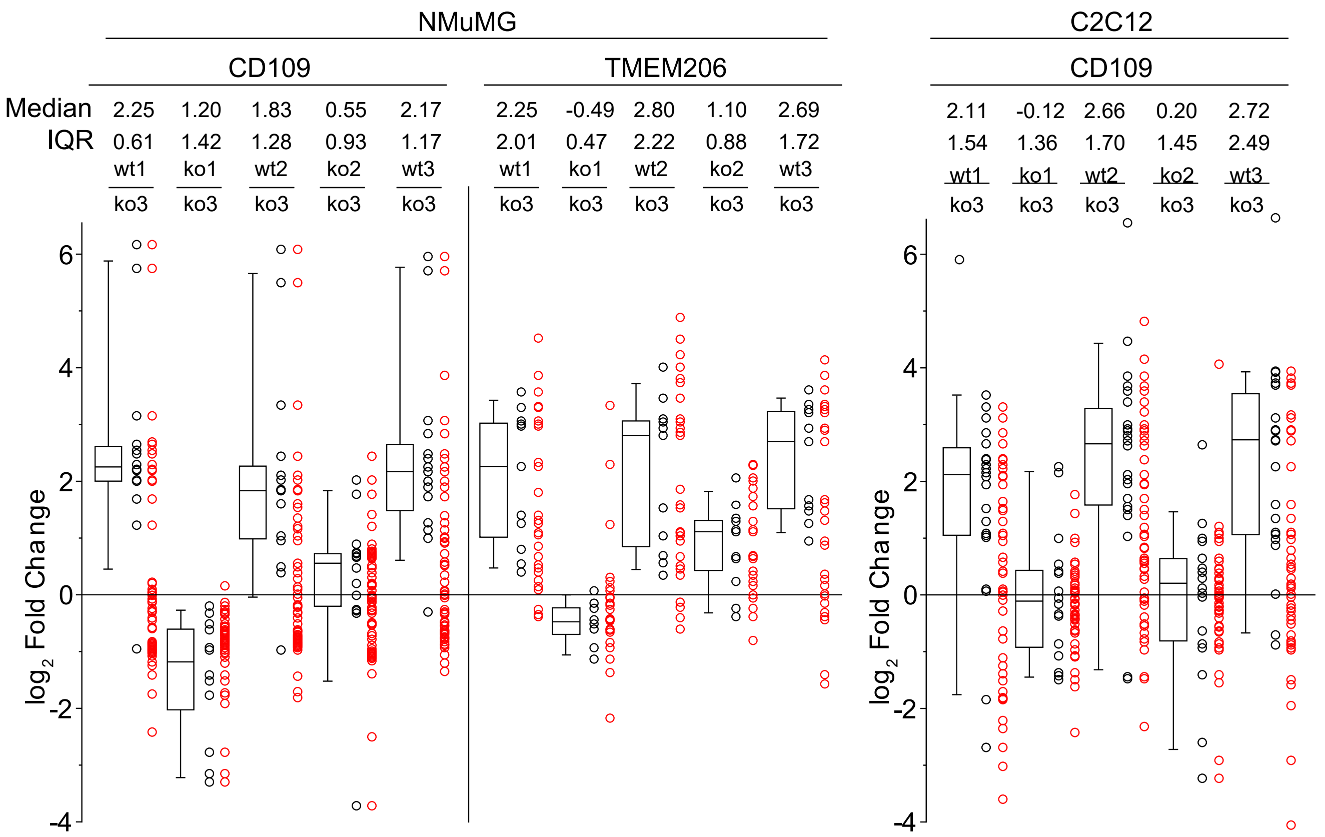

Supplementary Figure S3

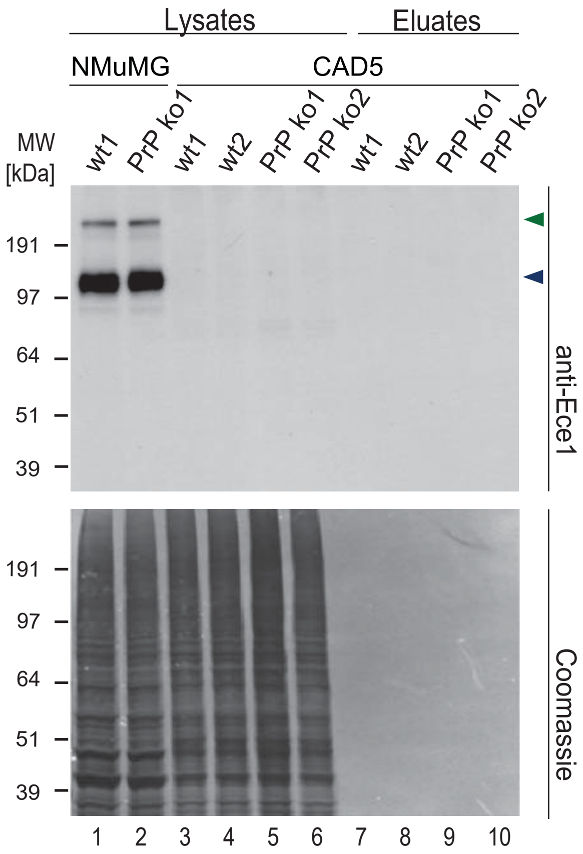

Supplementary Figure S4

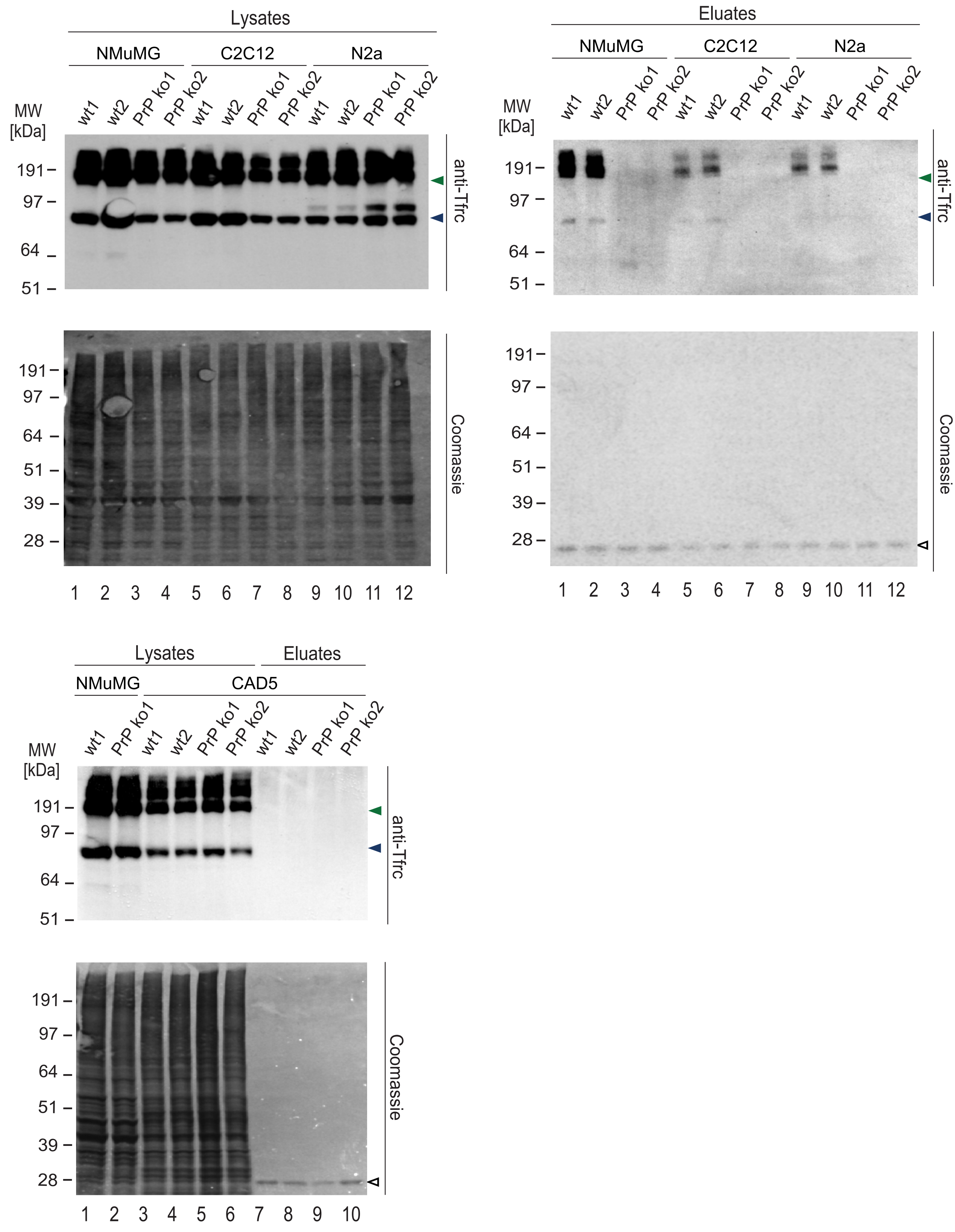

Supplementary Table S1. Comparison of the PrP interactome in four mouse cell models (only a subset of non-specific interactors shown)

						NMuMG							C2C12							N2A							CAD5					
Accession	Protein name	Gene	Coverage	Peptides	PSMs	wt1 ko3	ko1 ko3	wt2 ko3	ko2 ko3	wt3 ko3	Count	Score	wt1 ko3	ko1 ko3	wt2 ko3	ko2 ko3	wt3 ko3	Count	Score	wt1 ko3	ko1 ko3	wt2 ko3	ko2 ko3	wt3 ko3	Count	Score	wt1 ko3	wt2 ko3	wt3 ko3	ko2 ko3	Count	Score
IPI00120793.1	Major prion protein	Prnp	62.99%	12	957	12.75	0.80	16.29	1.14	11.43	49	558.5	22.02	1.59	27.84	2.95	30.59	55	512.1	10.88	1.38	8.35	1.70	7.63	60	371.9	13.37	17.31	13.89	0.67	38	365.3
IPI00122971.2	Neural cell adhesion molecule 1	Ncam1	75.52%	66	852	4.62	0.75	3.97	2.02	4.88	24	149.6	10.26	1.52	4.76	1.01	6.61	6	49.9	13.72	1.60	17.55	1.18	21.13	86	536.8	10.05	13.20	12.00	0.84	103	897.8
IPI00273801.3	Zinc transporter ZIP10	Slc39a10	24.37%	18	32																						32.77	44.87	43.18	0.23	2	30.9
IPI00930882.1	4F2 cell-surface antigen heavy chain	Slc3a2	61.77%	35	495	2.44	1.15	1.52	0.90	5.28	2	21.0								7.07	1.26	9.69	1.01	10.55	83	438.8	12.54	14.78	11.53	1.08	43	333.3
IPI00129395.2	Large neutral amino acids transporter small subunit 1	Slc7a5	36.13%	10	33															6.88	1.34	7.69	1.35	7.81	9	35.2	18.78	18.62	12.14	1.18	3	38.0
IPI00408495.1	Basigin	Bsg	56.04%	16	108															3.23	1.74	2.49	1.03	2.40	10	55.5	17.37	14.40	18.57	0.72	17	100.1
IPI00762815.1	Endothelin-converting enzyme 1	Ece1	63.89%	53	532	9.43	0.67	9.11	1.43	8.59	127	659.9	5.37	1.21	6.12	1.12	6.12	8	112.8													
IPI00947629.1	Thrombospondin type-1 domain-containing protein 7A	Thsd7a	45.57%	61	191							34.5															14.07	14.14	14.44	0.81	15	152.1
IPI00114022.1	Insulin-like growth factor-binding protein 5	Igfbp5	40.59%	10	33								9.67	2.42	9.37	3.02	12.96	5	18.1													
IPI00153809.1	CD109 antigen	Cd109	63.87%	77	535	4.74	0.44	3.56	1.47	4.49	15	294.0	4.32	0.92	6.32	1.15	6.61	22	348.1													
IPI00127983.1	Transmembrane emp24 domain-containing protein 2	Tmed2	40.80%	9	62	9.10	1.16	7.38	1.17	13.38	9	45.9																				
IPI00133522.2	Protein disulfide-isomerase	P4hb	65.42%	38	745	1.93	0.78	2.04	1.32	2.20	216	962.0	3.44	1.40	2.89	1.25	3.18	38	221.8	1.42	1.00	1.15	1.02	1.09	33	209.5	1.99	2.30	2.55	1.08	13	69.4
IPI00929762.1	Endoglin	Eng	71.61%	29	94															5.72	0.90	7.82	1.31	9.56	3	78.2						
IPI00466570.4	Transmembrane emp24 domain-containing protein 10	Tmed10	31.96%	6	23	6.34	1.09	6.25	1.03	9.59	16	76.1																				
IPI00311682.5	Sodium/potassium-transporting ATPase subunit alpha-1	Atp1a1	51.32%	46	319							34.0	1.35	2.57	0.96	1.27	0.65	2	31.6	2.80	1.25	2.90	0.68	2.57	15	201.7	3.50	3.29	3.65	0.97	20	274.0
IPI00132474.3	Integrin beta-1	Itgb1	47.74%	30	60	2.91	1.02	3.25	1.26	3.50	1	15.3								1.01	0.78	1.26	0.94	1.39	1	20.2	1.81	2.77	3.44	0.99	3	34.8
IPI00229517.5	Galectin-1	Lgals1	77.78%	11	459	1.38	0.89	1.20	1.33	1.83	126	493.1	1.67	1.06	1.57	1.13	1.46	58	271.1	2.04	1.39	1.40	0.71	0.90	31	93.5	1.97	2.85	2.71	0.94	12	
IPI00403079.4	Leukocyte surface antigen CD47	Cd47	40.26%	15	54																						5.70	6.11	8.11	0.53	2	54.7
IPI00187289.2	Transmembrane protein 206	Tmem206	85.14%	25	168	4.75	0.71	6.96	2.14	6.46	11	108.6																				
IPI00137336.1	Glypican-1	Gpc1	67.50%	34	164								4.54	1.20	5.26	1.12	6.24	20	204.9													
IPI00123639.1	Calreticulin	Calr	45.91%	13	93	1.47	0.92	1.25	0.98	1.71	5	44.0								1.57	0.96	1.65	0.91	1.51	10	86.8	1.50	1.95	1.75	0.96	4	29.1
IPI00272690.2	Angiotensin-converting enzyme	Ace	50.38%	58	475																						4.03	5.43	4.36	0.77	56	576.0
IPI00473680.2	Transmembrane emp24 domain-containing protein 9	Tmed9	42.75%	12	44	4.02	0.69	4.01	1.03	5.26	8	60.0																				
IPI00515173.1	H-2 class I histocompatibility antigen, K-K alpha	H2-K1	63.61%	23	78															3.41	1.04	4.49	1.29	5.04	5	35.2						
IPI00118413.2	Thromobospondin-1	Thbs1	37.92%	40	96	3.79	0.91	2.67	0.92	3.07	10	75.0																				
IPI00985828.1	H-2 class I histocompatibility antigen, D-P alpha	H2-D1	62.50%	29	140																						2.87	4.21	2.32	0.96	3	53.1
IPI00124700.1	Transferrin receptor protein 1	Tfrc	58.72%	50	289	2.09	0.76	2.02	1.43	2.06	31	186.0																				
IPI00972920.1	40S ribosomal protein S23	Rps23	55.24%	13	106	1.12	1.15	0.80	0.99	1.50	12	31.7	1.13	1.23	1.49	0.72	0.32	1	2.3	2.37	1.39	1.48	0.86	0.86	32	99.9	3.50	2.78	2.28	0.87	10	32.8
IPI00230660.5	40S ribosomal protein S15a	Rps15a	76.92%	9	89	1.11	1.87	0.51	1.24	2.14	14	52.7	0.94	0.93	0.96	0.87	0.55	12	27.5	1.61	1.62	0.84	0.74	0.46	14	42.5	2.86	4.19	2.55	1.00	3	
IPI00125901.5	40S ribosomal protein S13	Rps13	83.44%	20	85								1.01	0.93	1.18	1.38	0.97	6	34.6	1.39	1.32	1.19	0.79	0.56	15	69.7	4.53	4.50	2.78	1.13	4	21.3
IPI00986371.1	60S ribosomal protein L27a	Rpl27a	63.80%	13	49	1.22	1.41	0.56	0.89	1.84	9	36.2	0.66	0.35	0.34	0.22	0.51	4	21.0								4.94	1.56	5.58	1.35	2	7.8
IPI00988101.1	40S ribosomal protein S2	Rps2	68.26%	31	260	1.07	1.44	0.55	0.87	1.78	19	86.3	1.04	1.01	0.89	1.13	0.77	12	50.4	1.54	1.33	1.19	0.75	0.70	40	165.6	3.00	2.09	1.58	0.78	10	52.5
IPI00553538.3	Histone H3.1	Hist1h3a	73.53%	13	1061	1.17	1.78	0.30	0.73	2.51	79	388.4	4.74	1.98	1.45	1.60	0.55	27	337.3	2.83	1.71	1.43	0.42	0.34	152	795.3	0.65	1.28	1.21	1.04	18	96.9
IPI00876549.2	Histone H2B	Hist1h2b	79.85%	16	1365	1.64	1.77	0.34	0.72	2.94	81	327.9	3.59	2.59	1.16	1.65	0.38	58	379.9	3.16	1.60	1.67	0.51	0.46	264	1109.7	0.54	0.93	1.03	0.70	22	108.6
IPI00785343.2	Histone H3.3	H3f3a	72.79%	12	1076	1.10	1.77	0.29	0.73	2.29	82	437.6	4.37	1.96	1.70	2.19	0.36	38	399.9	2.74	1.75	1.41	0.42	0.34	148	762.9	0.65	1.28	1.21	1.04	18	99.4
IPI00282848.1	20 kDa protein	Hist1h3e	69.06%	18	1489	1.17	1.83	0.30	0.73	2.52	119	634.5	3.60	1.92	1.56	2.04	0.35	45	654.8	2.98	1.73	1.37	0.42	0.33	222	1341.4	0.65	1.28	1.21	1.02	19	105.6
IPI00407339.7	Histone H4	Hist1h4a	70.87%	15	1317	1.04	1.79	0.38	0.63	2.03	123	447.6	4.40	1.59	1.41	1.51	0.38	93	496.9	3.10	1.69	1.33	0.46	0.28	344	1379.5	0.62	1.22	1.10	0.95	44	213.8
IPI00230264.5	Histone H2A.x	H2afx	93.71%	24	746	1.10	1.56	0.33	0.73	2.20	48	157.1	2.54	1.95	1.76	2.04	0.55	35	216.3	1.97	1.68	1.05	0.56	0.29	183	757.8	0.71	1.28	1.20	1.01	14	61.1
IPI00117348.4	Tubulin alpha-1B chain	Tuba1b	69.18%	30	720	1.40	1.06	1.02	1.23	1.86	74	338.0	1.50	1.52	1.05	1.29	1.02	16	109.6	1.06	1.26	0.78	0.86	0.46	128	612.6	1.01	1.25	1.16	1.04	80	367.2
IPI00110753.1	Tubulin alpha-1A chain	Tuba1b	69.40%	30	721	1.38	1.05	1.02	1.22	1.83	68	319.5	1.31	1.38	1.13	1.38	0.89	22	140.7	0.97	1.25	0.73	0.88	0.44	120	588.5	1.05	1.24	1.14	1.04	84	392.4
IPI00112251.1	Tubulin beta-3 chain	Tuba1a	79.78%	33	636	1.48	1.09	0.86	1.19	1.93	49	227.9	0.95	1.14	0.92	1.03	0.92	26	146.4	0.90	1.26	0.71	0.83	0.39	108	579.4	1.07	1.22	1.09	1.03	48	248.4
IPI00117352.1	Tubulin beta-5 chain	Tubb5	79.95%	37	970	1.37	1.03	0.87	1.24	1.91	91	394.2	0.90	1.14	0.89	1.03	0.91	32	183.1	1.02	1.29	0.79	0.82	0.46	170	848.0	1.04	1.18	1.10	1.02	81	380.0
IPI00109061.1	Tubulin beta-2B chain	Tubb2b	79.78%	36	779	1.37	1.02	0.86	1.25	1.93	77	352.3	0.90	1.14	0.89	1.03	0.91	32	179.6	0.96	1.27	0.75	0.83	0.44	130	695.8	1.06	1.23	1.07	1.01	56	279.4
IPI00169463.1	Tubulin beta-2C chain	Tubb2c	73.71%	34	728	1.39	1.08	0.86	1.19	1.84	67	310.2	0.92	1.20	0.85	1.09	0.83	20	122.6	1.00	1.29	0.76	0.84	0.45	116	642.2	1.02	1.04	1.03	0.95	50	255.6
IPI00118384.1	14-3-3 protein epsilon	Ywhae	64.31%	19	333	0.79	1.35	0.50	0.85	1.37	55	212.7	1.63	4.20	0.94	1.19	0.73	32	144.4	1.54	1.61	0.79	0.55	0.37	35	143.6	1.14	1.47	1.26	0.87	3	13.6
IPI00227392.5	14-3-3 protein eta	Ywhah	47.97%	15	338	0.81	1.33	0.52	0.86	1.39	49	168.5	1.47	4.24	0.89	1.13	0.68	27	109.6	1.54	1.61	0.80	0.55	0.37	41	179.6	1.14	1.47	1.26	0.87	3	13.6
IPI00230707.6	14-3-3 protein gamma	Ywhag	59.11%	20	410	0.79	1.33	0.52	0.92	1.35	58	205.6	1.45	4.16	0.89	1.13	0.68	29	127.2	1.50	1.54	0.78	0.59	0.37	65	315.7	1.14	1.47	1.38	1.22	5	13.6
IPI00754545.3	14-3-3 protein theta-like	Ywhaq	81.82%	29	406	0.81	1.33	0.53	0.89	1.41	56	266.4	1.46	4.20	0.89	1.11	0.68	28	128.8	1.53	1.59	0.82	0.57	0.37	43	205.4	1.04	1.36	1.24	0.88	4	40.7
IPI00116498.1	14-3-3 protein zeta/delta	Ywhaz	79.59%	26	627	0.87	1.29	0.52	0.88	1.47	114	532.6	1.36	2.16	0.75	1.11	0.68	62	329.1	1.52	1.51	0.84	0.56	0.37	68	275.9	0.95	1.27	1.22	0.68	5	13.6

Colors shading as for Table 1. In addition, grey shading of different intensities was used to visually separate non-specific interactors belonging to distinct protein families.

Supplementary Table S2. Global proteome analysis of NMuMG cells -/+ TGFB1 (datset I) in PrP-deficient and wt cells (dataset II)

			Global proteome analyses / NMuMG cells												TMT Averages	
			dataset I						dataset II						I	II
Accession	Modified description	Coverage	-TGFB1 / +TGFB1	+TGFB1 / +TGFB1	-TGFB1 / +TGFB1	+TGFB1 / +TGFB1	-TGFB1 / +TGFB1	Count	PrP kd1 / wt3	wt1 / wt3	PrP kd2 / wt3	wt2 / wt3	PrP kd3 / wt3	Count	-/+TGFB1	wt
IPI00230665.3	Neural cell adhesion molecule 1 isoform 3	73.27%	0.367	0.989	0.353	0.943	0.378	28	0.762	0.986	0.757	0.944	0.751	57	0.366	0.757
IPI00132474.3	Integrin beta-1	75.56%	0.627	0.963	0.667	0.958	0.614	20	1.022	0.974	1.004	1.009	0.993	27	0.636	1.006
IPI00118413.2	Thrombospondin-1	67.55%	0.598	0.963	0.632	0.807	0.716	3	0.989	0.951	1.007	0.887	1.000	10	0.649	0.999
IPI00229517.5	Galectin-1	96.30%	0.826	0.919	0.802	1.138	0.796	35	1.047	0.963	1.133	1.054	1.156	41	0.808	1.112
IPI00124700.1	Transferrin receptor protein 1	74.31%	0.890	0.949	0.961	0.972	0.968	37	0.956	1.004	0.962	0.937	0.895	36	0.940	0.938
IPI00403079.4	Leukocyte surface antigen CD47	61.06%	1.169	1.103	1.101	1.027	1.082	12	1.051	1.023	0.967	0.965	1.018	13	1.117	1.012
IPI00311682.5	Sodium/potassium-transporting ATPase subunit alpha-	61.68%	1.314	1.020	1.358	1.043	1.350	89	0.926	1.025	0.929	1.014	0.939	76	1.341	0.931
IPI00930882.1	4F2 cell-surface antigen heavy chain isoform a	76.46%	1.182	1.044	1.209	0.956	1.177	23	1.027	1.060	0.968	1.005	1.063	22	1.189	1.019
IPI00129395.2	Large neutral amino acids transporter small subunit 1	42.58%	1.514	1.123	1.438	1.071	1.425	11	1.121	1.101	1.023	1.200	1.165	9	1.459	1.103
IPI00133522.2	Protein disulfide-isomerase	96.86%	0.929	1.017	0.935	0.990	0.908	82	0.883	0.998	0.898	1.012	0.914	104	0.924	0.898
IPI00123639.1	Calreticulin	73.32%	0.987	0.950	0.961	1.071	0.964	52	0.935	0.964	0.952	1.001	0.978	34	0.971	0.955
IPI00127983.1	Transmembrane emp24 domain-containing protein 2	71.14%	0.890	0.919	0.867	0.899	0.904	13	0.939	1.055	0.981	0.910	1.010	12	0.887	0.977
IPI00473680.2	Transmembrane emp24 domain-containing protein 9	83.64%	0.944	0.998	1.000	0.982	0.885	15	0.893	0.963	0.980	1.051	1.055	16	0.943	0.976
IPI00466570.4	Transmembrane emp24 domain-containing protein 10	62.10%	0.980	0.998	0.943	1.009	0.934	12	1.004	0.982	0.893	0.970	0.985	25	0.952	0.961

Heat map color code

proteins with relatively high levels of expression in:

dataset I — wt / wt + TGFB1 — epithelial wt cells ↔ mesenchymal wt cells

dataset II — PrP kd +TGFB1 / wt + TGFB1 — mesenchymal stable PrP kd cells ↔ mesenchymal wt cells
